# Molecular basis of product recognition during PIP5K-mediated production of PI(4,5)P_2_ with positive feedback

**DOI:** 10.1016/j.jbc.2024.107631

**Published:** 2024-08-03

**Authors:** Benjamin R. Duewell, Katherine A. Faris, Scott D. Hansen

**Affiliations:** 1 Department of Chemistry and Biochemistry, University of Oregon, Eugene, Oregon, USA; 2 Institute of Molecular Biology, University of Oregon, Eugene, Oregon, USA

**Keywords:** phosphatidylinositol phosphate lipids, PIP lipids, PIP5K, kinase, PI(4,5)P_2_

## Abstract

The ability for cells to localize and activate peripheral membrane-binding proteins is critical for signal transduction. Ubiquitously important in these signaling processes are phosphatidylinositol phosphate (PIP) lipids, which are dynamically phosphorylated by PIP lipid kinases on intracellular membranes. Functioning primarily at the plasma membrane, phosphatidylinositol-4-phosphate 5-kinases (PIP5K) catalyzes the phosphorylation of PI(4)P to generate most of the PI(4,5)P_2_ lipids found in eukaryotic plasma membranes. Recently, we determined that PIP5K displays a positive feedback loop based on membrane-mediated dimerization and cooperative binding to its product, PI(4,5)P_2_. Here, we examine how two motifs contribute to PI(4,5)P_2_ recognition to control membrane association and catalysis of PIP5K. Using a combination of single molecule TIRF microscopy and kinetic analysis of PI(4)P lipid phosphorylation, we map the sequence of steps that allow PIP5K to cooperatively engage PI(4,5)P_2_. We find that the specificity loop regulates the rate of PIP5K membrane association and helps orient the kinase to more effectively bind PI(4,5)P_2_ lipids. After correctly orienting on the membrane, PIP5K transitions to binding PI(4,5)P_2_ lipids near the active site through a motif previously referred to as the substrate or PIP-binding motif (PIPBM). The PIPBM has broad specificity for anionic lipids and serves a role in regulating membrane association *in vitro* and *in vivo*. Overall, our data supports a two-step membrane-binding model where the specificity loop and PIPBM act in concert to help PIP5K orient and productively engage anionic lipids to drive the positive feedback during PI(4,5)P_2_ production.

Phosphatidylinositol phosphate (PIP) lipids are important second messengers that function as molecular scaffolds for numerous membrane-binding proteins. PIP lipids are dynamically interconverted between seven different species by specialized kinases and phosphatases (1). Research has demonstrated that each PIP lipid species exists in spatially distinct subcellular membranes and function to recruit specific enzymes to their necessary location for proper signaling (2, 3). The most abundant PIP lipid that mediates signaling at the plasma membrane is phosphatidylinositol-4,5-bisphosphate (PI(4,5)P_2_), comprising 2 to 5% of total plasma membrane lipids (4, 5, 6). Unlike most PIP lipids, PI(4,5)P_2_ is omnipresent at the plasma membrane and is critical for several constitutive cellular functions, including cytoskeletal assembly (7, 8), ion channel gating (9, 10), and endocytosis (11). Without a constant and stable pool of PI(4,5)P_2_, these cellular signaling events are hindered and cells adopt disease-like states (12).

Phosphatidylinositol-4-phosphate 5-kinase (PIP5K) family of enzymes are responsible for creating the bulk of PI(4,5)P_2_ lipids in eukaryotic cells (13). PIP5K enzymes function by binding their substrate, phosphatidylinositol-4-phosphate (PI(4)P), then catalyzing the phosphorylation of the 5-hydroxyl group on the inositol lipid head group in the presence of Mg^(+2)^-ATP (14, 15). This activity has been described as specific to the PI(4)P substrate, although PIP5K can also phosphorylate the 5-OH of other PIP lipid species with lower efficiency (16, 17, 18, 19, 20). In mammals, PIP5K exists as three paralogs (*i.e.* α, β, and γ) that display tissue-specific expression patterns (21, 22, 23). Localization studies in macrophages have shown that PIP5K paralogs predominately localize to the plasma membrane (24). PIP5K has been implicated in many cellular pathways, with specific regulatory importance in the Wnt pathway (25), phagocytosis (26, 27), and focal adhesions (28, 29). More recently, medical studies have begun to target PIP5K with potential as a cancer therapeutic (30). Studies that have knocked out PIP5K from both prostate and breast cancer models showed diminished tumorigenesis and metastatic properties (31). The drugs designed to inhibit PIP5KA activity show promise reducing tumor growth and proliferation; however, these drugs have low specificity for PIP5K (32). As our understanding of PIP5K’s role in tumor proliferation evolves, it is important that we continue to decipher the molecular mechanisms that regulate PIP5K membrane localization and activity in cells.

Recently, our lab established a supported lipid bilayer (SLB) and total internal reflection fluorescence microscopy (TIRF-M) assay to directly visualize the membrane-binding dynamics of purified PIP5K with single molecule resolution *in vitro*. We previously reported that human PIP5KB binds cooperatively to its product, PI(4,5)P_2_, which enhances PIP5K membrane localization through a PI(4,5)P_2_-mediated positive feedback loop (33). Positive feedback is based on an increased affinity of PIP5K for PI(4,5)P_2_ compared to PI(4)P lipids (Fig. S1), leading to a greater density of PIP5K on the membrane as PI(4,5)P_2_ is generated. Further, we have shown that when PIP5K crosses a threshold membrane surface density of ∼ 100 molecules/μm^2^, it can dimerize with another membrane bound kinase, potentiating lipid kinase activity (34). Membrane-bound PIP5K has an increased likelihood to encounter PI(4)P lipids in a conformational state that is ideal for productive binding and catalysis, leading to an increased apparent activity of PIP5K enzymes. This suggests that the site of PI(4,5)P_2_ binding is an important step for plasma membrane localization in cells, as PI(4,5)P_2_ lipids exist almost exclusively at the plasma membrane. Currently, the residues or motif(s) that regulates high affinity PI(4,5)P_2_ binding remains unknown.

To date, much work has been done to define PIP5Ks enzymatic activity, including identifying motifs that regulate substrate specificity, ATP binding, and hydrolysis (17, 18, 35). Structural biochemistry has helped elucidate the mechanism controlling PIP5K substrate recognition (18, 35). X-ray crystallography of zebrafish PIP5K (zPIP5K) determined that PIP5K binds to its substrate, PI(4)P, through the DLKGSxxxR motif, which is also referred to as the PI(4)P or substrate-binding motif (18, 36). Mutations in the PI(4)P-binding motif (PIPBM) of PIP5Ks abolishes lipid kinase activity (18). PIP5K substrate specificity has been found to be mediated by a structural motif referred to in the literature as either the activation loop or specificity loop. Structural analysis of zPIP5KA using NMR suggests that the specificity loop exchanges between a disordered and an alpha helical conformation (35). Membrane–lipid interactions are hypothesized to shift the equilibrium to favor the alpha helical conformation, allowing hydrophobic residues in the specificity loop to insert into a lipid bilayer (35). Mutating lysine residues in the specificity loop have been shown to disrupt plasma membrane localization (16). Mutations that break the alpha helical character, but preserve the overall basic charge, have also been shown to disrupt lipid kinase activity (35). In both cases, loss of PIP5K function has been attributed to perturbing the PI(4)P-binding interaction. While these studies have revealed which motifs are critical for PIP5K lipid kinase activity, we do not currently understand how these motifs contribute to the cooperative PI(4,5)P_2_ binding, membrane-mediated dimerization, and positive feedback. Deciphering these mechanisms could help explain why PIP5K is predominantly localized and activated at the plasma membrane.

Here we present a mutational analysis and single molecule TIRF-M study of PIP5K, which reveals the sequence of molecular interactions that control PIP5K membrane docking and PI(4,5)P_2_ engagement. The initial membrane interaction is regulated by the specificity loop, which plays an important first step for membrane binding but is not essential for productive docking on PI(4,5)P_2_-containing membranes. Using a set of chimeric proteins that contain different specificity loops, we show that the initial binding step of PIP5K can be mediated by interactions with either PI(4)P or PI(4,5)P_2_ lipids. Our findings are consistent with the previously reported substrate specificity for PIP5K. Interactions with the lipid product, PI(4,5)P_2_, were also paradoxically mediated through a region of the kinase commonly referred to as the PIPBM or substrate-binding motif. We discovered that the PIPBM is a broad anionic lipid sensor that can associate with lipids including phosphatidylserine (PS) and all types of PIP lipids. Overall, the strength of lipid interactions with the PIPBM is strongly correlated with the valency of phosphorylation on the PIP lipid head group, with triply phosphorylated PIP lipids exhibiting the strongest interaction. Supporting that the PIPBM controls membrane localization in addition to catalysis, we find that PIPBM mutants can still phosphorylate PI(4)P when localized to bilayers artificially. Finally, we used new insight about PIP5Ks’ membrane-binding mechanism to validate separation of function mutants that are catalytically dead but retain their ability to localize to PI(4,5)P_2_-containing membranes. Overall, this work improves our understanding of the molecular mechanisms underlying PI(4,5)P_2_-mediated recruitment of PIP5K to membranes.

## Results

### The specificity loop regulates PIP5K localization to PI(4,5)P_2_-containing membranes

**Figure 1:**
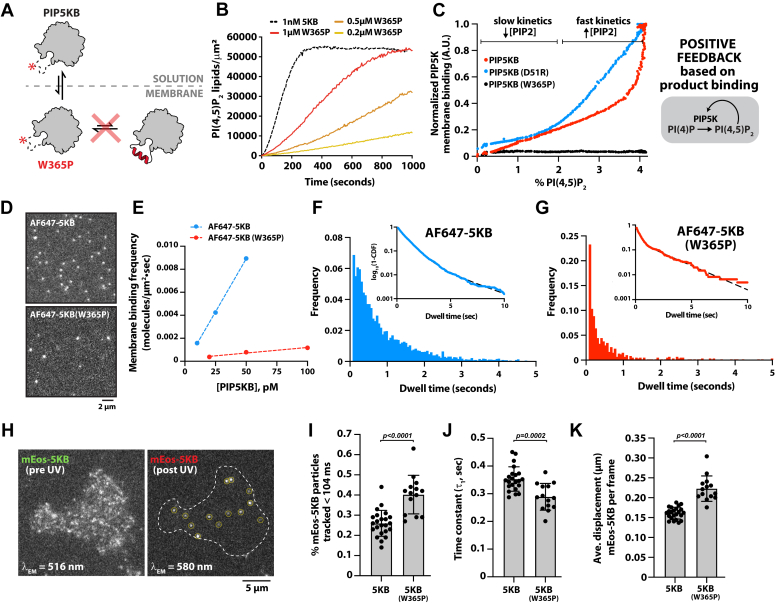
**ThePIP5Kspecificity loop regulates the membrane association and dissociation rate constants.***A*, cartoon illustrating how the W365P mutation disrupts the specificity loop alpha helical conformation in PIP5KB. *B*, representative lipid kinase activity traces measured in the presence of PIP5KB and PIP5KB (W365P). Production of PI(4,5)P_2_ was monitored in the presence of 20 nM Cy3-PLC*δ* and the indicated concentration of PIP5KB. *C*, PIP5KB (WT and D51R) but not PIP5KB (W365P) display cooperative PI(4,5)P_2_-dependent changes in supported membrane localization. Phosphorylation of PI(4)P and membrane localization of PIP5KB were monitored in the presence of 5 nM AF647-PIP5KB, 5 nM mNG-PIP5KB (D51R), or 5 nM AF647-PIP5KB (W365P). The production of PI(4,5)P_2_ was visualized using 20 nM Cy3-PLC*δ* in each kinase reaction. *B* and *C*, initial membrane composition: 96% DOPC, 4% PI(4)P. *D*, representative TIRF-M images showing membrane localization in the presence of 100 pM AF647-PIP5KB or AF647-PIP5KB (W365P). *E*, experimental measure of membrane-binding frequency in the presence of 10 to 50 pM AF647-PIP5KB (*k*_*ON*_ = 184/μM⋅μm^2^⋅sec) and 20 to 100 pM AF647-PIP5KB (W365P, *k*_*ON*_ = 9.68/μM•μm^2^•sec). *F* and *G*, single molecule dwell time distributions measured in the presence of either AF647-PIP5KB (τ_1_ = 0.815 ± 0.054s, τ_2_ = 3.09 ± 0.35 s, α = 0.95 ± 0.014, n = 14,950) or AF647-PIP5KB (W365P) (τ_1_ = 0.296 ± 0.11s, τ_2_ = 2.82 ± 0.71s, α = 0.77 ± 0.056, n = 2389). *D*–*G*, membrane composition: 98% DOPC, 2% PI(4,5)P_2_. Errors equal SD from n ≥ 3 technical replicates. *H*, representative TIRF-M images of mEos-PIP5KB plasma membrane localization before and after photoconversion with 405 nm light in HEK293T cells. *I*, a larger fraction of mEos-PIP5KB W365P (40%) display transient membrane-binding events < 104 ms, compared to the WT mEos-PIP5KB (26%). Each data point equals the average number of transient molecules detected per cell (*N* = 14–23 cells). *J*, the mean dwell time of mEos-PIP5KB (W365P) (τ_1_ = 0.289 ± 0.05 s, *N* = 14 cells, *n* = 5662 molecules) is shorter than mEos-PIP5KB (τ_1_ = 0.353 ± 0.04 s, *N* = 23 cells, *n* = 19,087 molecules). Each data point is derived from a single exponential fit to a dwell time distribution from a single cell. *I* and *J*, each data point equals the average number of transient molecules or time constant (τ_1_, sec) calculated per cell (N = 14–23 cells). *K*, mEos-PIP5KB (W365P) is more diffuse in the cellular plasma membrane than mEos-PIP5KB. Average single molecule displacement during 52 ms time interval is greater for mEos-PIP5KB W365P (0.223 ± 0.03 μm, *N* = 14 cells) than mEos-PIP5KB (0.161 ± 0.01 μm, *N* = 23 cells). *I*–*K*, errors equal SD. Each data point is the average displacement measured for a single cell. *p* value calculated by unpaired two-tailed Student’s *t* test.

Previous studies indicate that PIP5K substrate recognition is regulated by the specificity loop (16, 17, 18, 35). Positioned within ∼20 Å of the active site, the specificity loop is thought to help organize the active site to orient the substrate, PI(4)P, for catalysis (18). NMR experiments also suggest that membrane docking of PIP5K causes the unstructured specificity loop to adopt an alpha helical conformation, which reportedly inserts into lipid membranes (35). Membrane insertion of an amphipathic helix is predicted to significantly enhance the membrane dwell time of PIP5K, which could contribute to the positive feedback mechanism involving cooperative membrane binding of PIP5K to the lipid product, PI(4,5)P_2_ (33) (Fig. S1). To determine how the specificity loop contributes to PIP5K localization and positive feedback, we disrupted alpha helix formation in the specificity loop by introducing a W365P mutation in PIP5KB (Fig. 1*A*). A homologous mutation (*i.e.* W393P) was previously shown to reduce the catalytic activity of zPIP5KA 25-fold relative to the WT kinase (35). Consistent with previous observations, PIP5KB (W365P) displayed a 50-fold reduction in lipid kinase activity compared to WT PIP5KB when measured on SLBs using TIRF microscopy (Fig. 1*B*). By contrast, the PIP5KB (W365A) mutant displayed a less dramatic decrease in catalytic activity (Fig. S2*A*), suggesting that preservation of the specificity loop alpha helical character and/or hydrophobicity is important for PIP5K catalysis. This is consistent with the zPIP5KA (W393G) mutant reportedly having similar kinase activity to WT zPIP5KA (35).

To determine the extent to which the reduced activity of PIP5KB (W365P) is due to defects in membrane localization, we used sortase-mediated peptide ligation to fluorescently label PIP5KB with Alexa Fluor 647 (AF647) and visualized the mutant kinase localization *in vitro* on reconstituted SLBs. Unlike WT and dimerization-deficient (*i.e.* D51R) AF647-PIP5KB, localization of AF647-PIP5KB (W365P) did not exhibit cooperative membrane recruitment that was coupled to the production of PI(4,5)P_2_ lipids (Figs. 1*C*, S1, *B* and *C*, and S2*B*). Overall, the reduced PIP5KB (W365P) activity was strongly correlated with a decrease in membrane localization.

Since membrane binding of AF647-PIP5KB (W365P) was unresponsive to the generation of PI(4,5)P_2_ lipids, we hypothesized that the specificity loop directly senses PI(4,5)P_2_ lipids, in addition to playing a critical role in substrate recognition. When we compared the single molecule localization of AF647-PIP5KB and AF647-PIP5KB (W365P), we observed a striking difference in the total number of membrane-binding events on PI(4,5)P_2_-containing bilayers (Fig. 1*D*). To quantify this difference, we measured the single molecule membrane-binding frequency of AF647-PIP5KB and AF647-PIP5KB (W365P) (Figs. 1*E* and S2, *C* and *D*). Mutating the specificity loop resulted in a 19-fold decrease in the association rate constant (*k*_*ON*_) compared to WT PIP5KB (Fig. 1*E*). A similar reduction in the membrane-binding frequency was observed for mNG-PIP5KB (W365A) (Fig. S2*E*). In addition to the observed reduction in *k*_*ON*_ for AF647-PIP5KB (W365P), disrupting the specificity loop reduced the single molecule dwell time in the presence of PI(4,5)P_2_ (Fig. 1, *F* and *G*). Considering that the specificity loop has been proposed to penetrate directly into membranes (35), we expected to observe a more dramatic decrease in the membrane dwell time. Mutations that preserve the alpha helical character of the specificity loop (*i.e.* W365A and L61E/W365R) but reduce the hydrophobicity displayed similarly modest decreases in the dwell time on PI(4,5)P_2_-containing membranes (Fig. S2*F*). Together, these findings suggest that while binding frequency and dwell time were diminished, PIP5K specificity loop mutants can still dock on PI(4,5)P_2_-containing membranes.

The single molecule supported membrane-binding experiments provide a useful platform to measure individual protein–lipid interactions; however, they lack some of the complexity of a cellular plasma membrane. For example, the supported bilayers described here lack sterol lipids and various peripheral membrane-binding proteins that could modulate membrane localization of PIP5K. To determine whether membrane binding of PIP5KB (W365P) was altered in living cells, we established conditions to visualize the plasma membrane localization of PIP5KB in human embryonic kidney (HEK)293T cells with single molecule resolution using TIRF-M. For these experiments, genes encoding either mEos-tagged PIP5KB or PIP5KB (W365P) were transiently expressed in HEK293T cells under a heat shock promoter, which yielded a near single molecule membrane density of fluorescently tagged kinase localized to the plasma membrane (Fig. 1*H*). Following a short pulse of 405 nm light, a fraction of the mEos-tagged PIP5K were photoconverted from the green to red fluorescent state, which provided the spatial resolution needed to track individual PIP5K molecules (Fig. 1*H*). Based on measurements across multiple cells, we classified significantly more transient plasma membrane localization events (*i.e.* < 104 ms) in the presence of mEos-PIP5KB (W365P) (Fig. 1*I*). Excluding these brief localization events from our data set, we still consistently measured average dwell times for mEos-PIP5KB (W365P) that were shorter than mEos-PIP5KB (Fig. 1*J*). Consistent with the W365P mutant having fewer membrane lipid interactions, the average single molecule displacement of mEos-PIP5KB (W365P) was significantly larger than mEos-PIP5KB (Fig. 1*K*). The observed membrane-binding frequency of mEos-PIP5KB (W365P) was also reduced. However, calculating the *k*_*ON*_ for mEos-PIP5KB (WT and W365P) in cells was impractical due to cell-to-cell variation in protein expression and differences in the fraction of photoconverted mEos, which make it challenging to estimate the total cellular concentration of mEos-PIP5K. Overall, our cellular measurements agree with our *in vitro* observations that the specificity loop facilitates PI(4,5)P_2_ binding but is not solely responsible for membrane localization.

### The PIP5K specificity loop enhances PI(4)P and PI(4,5)P_2_ specificity of chimeric proteins

**Figure 2:**
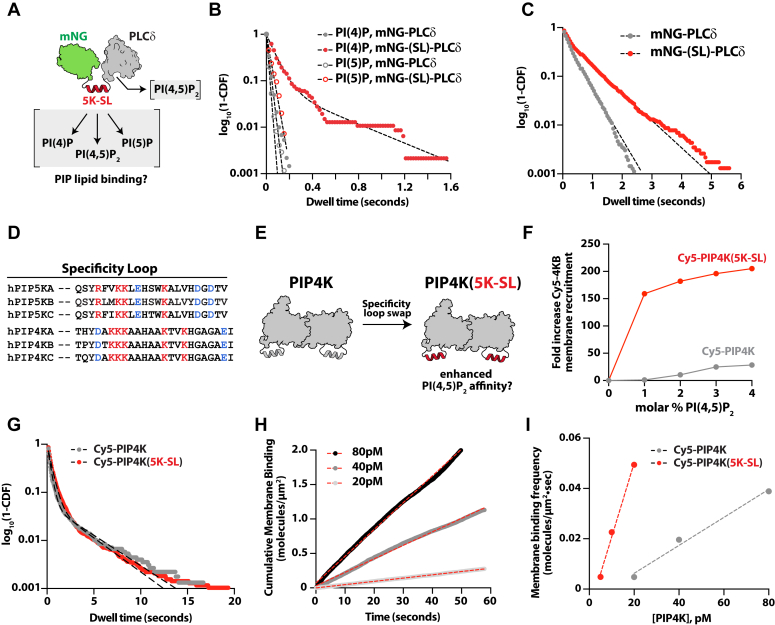
**The PIP5K specificity loop enhances PI(4,5)P**_**2**_**lipid binding.***A*, cartoon showing the structure of the mNG-PIP5K specificity loop-PLC*δ* chimeric protein. The expected PIP lipid-binding interactions are indicated. *B*, single molecule dwell time distributions of mNG-PLC*δ* measured on membranes containing either PI(4)P (τ_1_ = 20 ± 1 ms, n = 6456) or PI(5)P (τ_1_ = 26 ± 10 ms, n = 2872). Single molecule dwell time analysis of mNG-(5K-SL)-PLC*δ* yield the following time constants: PI(4)P (τ_1_ = 42 ± 34 ms, τ_2_ = 314 ± 271 ms, α = 0.81 ± 0.13, n = 4274) or PI(5)P (τ_1_ = 82 ± 13 ms, n = 855). In both cases, dwell times were measured on membrane containing 4% PI(4)P or 4% PI(5)P. *C*, single molecule dwell time distributions of mNG-PLC*δ* (τ_1_ = 0.346 ± 0.041 s, n = 5414) and mNG-(5K-SL)-PLC*δ* (τ_1_ = 0.127 ± 0.059 s, τ_2_ = 0.797 ± 0.066 s, α = 0.47 ± 0.06, n = 3082) measured on membranes containing 4% PI(4,5)P_2_. *D*, sequence alignment of PIP5K and PIP4K specificity loops. *E*, cartoon showing the PIP4K to PIP5K specificity loop swap. *F*, quantification of the fold increase in the localization of Cy5-PIP4K and Cy5-PIP4K(5K-SL) on membranes containing different molar concentrations of PI(4,5)P_2_. The fold change was calculated relative to the measured membrane density in the absence of PI(4,5)P_2_. *G*, single molecule dwell time distributions of Cy5-PIP4K (τ_1_ = 0.248 ± 0.021 s, τ_2_ = 1.82 ± 0.16 s, α = 0.81 ± 0.04, n = 13,117 molecules) and Cy5-PIP4K(5K-SL) (τ_1_ = 0.431 ± 0.067 s, τ_2_ = 8.77 ± 4.9 s, α = 0.84 ± 0.05, n = 12,720 molecules) measured on membranes containing 4% PI(4,5)P_2_. *H*, plots showing the cumulative membrane-binding events measured in the presence of 20 to 80 pM Cy5-PIP4K. The slope of each curve was calculated by linear regression yielding a binding frequency for each concentration of Cy5-PIP4K. *I*, experimental measure of membrane-binding frequency in the presence of 20 to 80 pM Cy5-PIP4K (*k*_*ON*_ = 0.56/nM⋅μm^2^⋅sec) or 5 to 10 pM Cy5-PIP4K(5K-SL) (*k*_*ON*_ = 2.93/nM⋅μm^2^⋅sec). *B,C, andG*, errors equal SD from n ≥ 3 technical replicates.

Previous research indicates that the specificity loop controls membrane docking of PIP5K through substrate specificity (16, 18, 35). This is supported by molecular dynamics simulations that indicate membrane docking relies on the initial engagement of PI(4)P by the specificity loop, which then transitions to the active site for catalysis (37). Based on our single molecule dwell time analysis of AF647-PIP5KB on SLBs, the specificity loop regulates the rate of membrane association (*k*_*ON*_) on PI(4,5)P_2_-containing membranes (Fig. 1*E*). Given these findings, we hypothesized that the specificity loop can directly interact with PI(4,5)P_2_ lipids, a function previously uncharacterized. One question that arises with this hypothesis is whether the specificity loop functions as a broad anionic lipid-binding domain or if it has specificity for certain PIP lipids. Given that the specificity loop was previously shown to favor PI(4)P lipids as a substrate for catalysis, we hypothesized that the specificity loop does not act as a broad anionic lipid-binding domain. Testing these hypotheses in the context of the full-length PIP5K protein, however, would be difficult to interpret due to the highly electro-positivity surface proposed to interact with anionic lipids. To isolate the role the specificity loop alone serves as a PI(4)P or PI(4,5)P_2_ lipid sensor, we created a chimeric mNeonGreen (mNG) fusion protein containing the PIP5K specificity loop fused to the pleckstrin homology (PH) domain derived from phospholipase C-*δ*1 (*i.e.* mNG-5K(SL)-PLC*δ*) (Fig. 2*A*). Characterization of the mNG-5K(SL)-PLC*δ* membrane-binding properties in the context of PLC*δ* provided an approach for measuring the potential gain in PIP lipid specificity and enhanced membrane-binding affinity due to the addition of the PIP5KB specificity loop. Using single molecule TIRF-M, we examined whether mNG-5K(SL)-PLC*δ* acquired membrane-binding specificity for either PI(4)P or PI(5)P lipids (Fig. 2*B*). Consistent with PH-PLC*δ* lacking specificity for either PI(4)P or PI(5)P, mNG-PLC*δ* did not strongly interact with either lipid on supported membranes (Fig. 2*B*). By contrast, we observed an increase in the single molecule dwell time for mNG-5K(SL)-PLC*δ* in the presence of PI(4)P, but not PI(5)P (Fig. 2*B*). This result suggests that the PIP5K specificity loop can discriminate between PI(4)P and PI(5)P, even when fused to a different peripheral membrane-binding protein. Note that we also attempted to visualize membrane interactions of mNG fused to the PIP5K specificity loop (*i.e.* mNG-5K-SL) alone; however, this chimeric protein displayed very transient membrane interactions that were not quantifiable. We assume that the additional weak electrostatic interactions provided by PH-PLC*δ* facilitate membrane association of 5K(SL). As a control, we performed similar single molecule tracking experiments on PI(4)P- and PI(5)P-containing membranes in the presence of both full length AF647-PIP5KB and the AF647-PIP5KB (W365P) specificity loop mutant (Fig. S3, *A*–*C*). Similar to the trends in Figure 2*B*, mutating the specificity loop had a more dramatic effect on the dwell times measured in the presence of PI(4)P compared to PI(5)P (Fig. S3, *B* and *C*).

In addition to regulating substrate recognition, our results indicate that the specificity loop controls PI(4,5)P_2_ lipid binding and contributes to the positive feedback loop observed during PIP5K-mediated production of PI(4,5)P_2_. To determine if the PIP5K specificity loop can generally enhance the affinity of other membrane-binding proteins, we compared the single molecule dwell times of mNG-PLC*δ* and mNG-5K(SL)-PLC*δ* on PI(4,5)P_2_-containing membranes (Fig. 2*C*). Both mNG-PLC*δ* and mNG-5K(SL)-PLC*δ* displayed transient dwell times that were nearly identical, due to both proteins sharing the common PH-PLC*δ* domain (Fig. 2*C*). Looking at the dwell time distribution of mNG-5K(SL)-PLC*δ*, we observed a second population of longer dwelling molecules, consistent with the specificity loop enhancing PI(4,5)P_2_ association (Fig. 2*C*).

Our recent membrane-binding study of the type II phosphatidylinositol-5-phosphate 4-kinase (PIP4K) revealed that a threshold membrane density of ∼3% PI(4,5)P_2_ lipids was required to observe robust PIP4K localization on SLBs (38). By contrast, PIP5K strongly localizes to membranes containing only 1% PI(4,5)P_2_ (33, 38). To determine whether the difference in PI(4,5)P_2_-dependent membrane association is related to the specificity loop sequence variation between PIP4K and PIP5K (Fig. 2*D*), we generated a PIP4KB to PIP5KB specificity loop swap referred to as PIP4K(5K-SL) (Fig. 2*E*). We quantified the difference in Cy5-PIP4K and Cy5-PIP4K(5K-SL) bulk membrane recruitment on supported membranes containing varying concentrations of PI(4,5)P_2_ (Figs. 2, *F* and *G* and S4). In the presence of 1% PI(4,5)P_2_, Cy5-PIP4K(5K-SL) reached a final equilibrium membrane surface density that was 160-fold greater than Cy5-PIP4K (Fig. 2*F*). Visualization of the single molecule membrane-binding properties of Cy5-PIP4K and Cy5-PIP4K(5K-SL) revealed an increase in the mean dwell time (0.56 and 0.66 s, respectively) due to the specificity loop (Fig. 2*G*). Given the extremely long dwell times displayed by Cy5-PIP4K and Cy5-PIP4K(5K-SL), we are potentially underestimating the total specificity loop–dependent increase in membrane dwell time due to Cy5 photobleaching. Consistent with the specificity loop enhancing PIP5K membrane localization to PI(4,5)P_2_-containing membranes, swapping the specificity loop also increased the association rate constant (*k*_*ON*_) of Cy5-PIP4K(5K-SL) by 5-fold (Fig. 2, *H* and *I*). Considering that the PIP4KB to PIP5KB specificity loop swap reduces the isoelectric from 9.7 to 8.4, the observed enhancement in membrane localization for Cy5-PIP4K(5K-SL) cannot be attributed solely to a change in net positive charge. Overall, these data confirm that the PIP5K specificity loop can sense both PI(4)P and PI(4,5)P_2_ lipids. This data also provides one explanation for why PIP4K has a reduced capacity to localize to PI(4,5)P_2_ lipids compared to PIP5K both *in vivo* and *in vitro* (38).

### Molecular basis of the PIP5K high affinity PI(4,5)P_2_ lipid interaction

**Figure 3:**
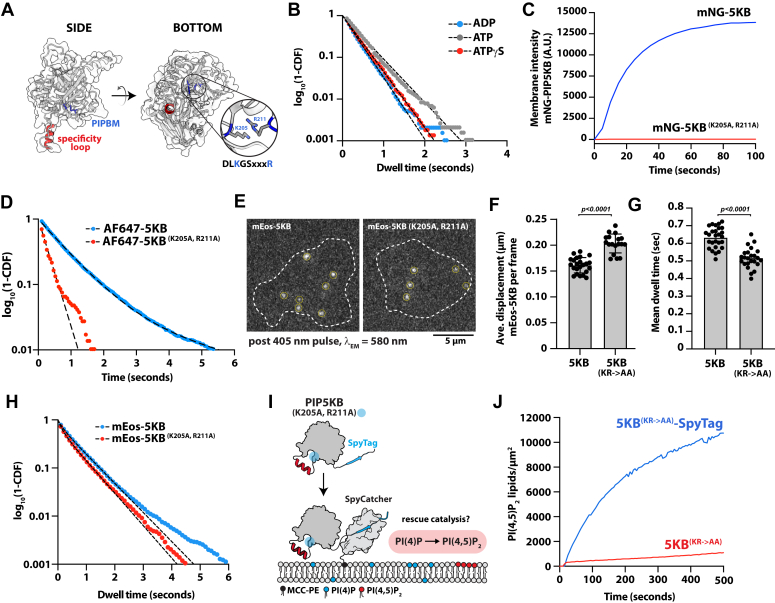
**PIP5K can engage PI(4,5)P**_**2**_**lipids through the substrate-binding motif.***A*, AlphaFold structure of monomeric human PIP5KB validated against zPIP5Kα (5E3U.pdb). Structure highlights the specificity loop (*red*) and the PIP lipid (or substrate) binding motif (PIPBM, *blue*). *B*, single molecule dwell time distributions of AF647-PIP5KB measured in the buffer containing 1 mM ATP (τ_1_ = 0.463 ± 0.16 s, n = 6377), 1 mM ADP (τ_1_ = 0.365 ± 0.11 s, n = 10,543), or 1 mM ATPγS (τ_1_ = 0.329 ± 0.004 s, n = 6231). Errors equal SD from n = 3 technical replicates. *C*, bulk membrane recruitment of either 25 nM mNG-PIP5KB or mNG-PIP5KB (K205A/R211A). *B* and *C*, membrane composition: 98% DOPC, 2% PI(4,5)P_2_. *D*, single molecule dwell time distribution comparing AF647-PIP5KB (τ_1_ = 0.815 ± 0.054, τ_2_ = 3.09 ± 0.35, α = 0.95 ± 0.01, n = 14950) and AF647-PIP5KB (K205A/R211A, τ_1_ = 0.306 ± 0.19 s, n = 684). Membrane composition: 98% DOPC, 2% PI(4,5)P_2_. *E*, representative TIRF-M images showing the localization of the mEos-PIP5KB (WT and K205A/R211A) expressed in HEK293T cells. *F*, mEos-PIP5KB (K205A, R211A) is more diffuse in the cellular plasma membrane than mEos-PIP5KB. Average single molecule displacement during 52 ms time interval is greater for mEos-PIP5KB K205A,R211A (0.203 ± 0.02 μm, *N* = 18 cells) than mEos-PIP5KB (0.161 ± 0.01 μm, *N* = 23 cells). Each data point is the average displacement measured for a single cell. *G*, the mean dwell time of mEos-PIP5KB K205A/R211A (0.516 ± 0.06 s, *N* = 24 cells) is shorter than mEos-PIP5KB (0.632 ± 0.06s, *N* = 27 cells). Each data point is derived from a single cell. *F* and *G*, *p* value calculated by unpaired Student’s *t* test. *H*, significantly more transient interactions observed for mEos-PIP5KB K205A/R211A (τ_1_ = 0.142 s, τ_2_ = 0.645 s, α = 0.34, n = 15,162 molecules, *N* = 24 cells) than mEos-PIP5KB (τ_1_ = 0.159 s, τ_2_ = 0.719 s, α = 0.22, n = 19450 molecules, *N* = 27 cells [α represents the fraction of short dwelling molecules (τ_1_)]). Dwell time distribution was fit using all single molecule-binding events measured across all cells. *I*, schematic showing SpyTag-PIP5KB (K205A/R211A) and SpyCatcher kinase activity assay. *J*, kinetics of lipid phosphorylation measured for membrane tethered and nontethered SpyTag-PIP5KB (K205A/R211A). Production of PI(4,5)P_2_ was monitored in the presence of 20 nM Cy3-PLC*δ*. Initial membrane composition: 98% DOPC, 2% PI(4)P. Errors equal SD.

Having established that the specificity loop serves an important role in regulating the initial membrane docking step of PIP5K, we sought to identify residues responsible for the high affinity PI(4,5)P_2_ lipid-binding interaction. We hypothesized the existence of this interaction site based on the ability of AF647-PIP5K (W365P) to stably associate with PI(4,5)P_2_-containing membranes (Fig. 1). PIP5K contains numerous basic amino acids on the membrane-binding interface that could promote interactions with anionic lipids (24, 39). Structural biochemistry studies of zPIP5KA protein crystals soaked with selenate resolved a SeO_4_^2-^ molecule bound to basic residues (*i.e.* K238 and R244) near the ATP-binding pocket (18). These residues are part of a conserved sequence, DLKGSxxxR, which is referred to as the PIPBM or substrate-binding motif (18) (Fig. 3*A*). The PIPBM is considered part of the kinase domain active site and plays an important role in positioning PI(4)P near the ATP γ-phosphate for efficient lipid phosphorylation (18). Consistent with this model, molecular dynamic simulations have shown that zPIP5KA interacts with PI(4)P lipids through the PIPBM (37). Mutation in R244 has been shown to reduce zPIP5KA lipid kinase activity but paradoxically does not affect the intrinsic ATP hydrolysis rate of zPIP5KA in solution (18).

Does the PIPBM regulate PI(4,5)P_2_ binding? Structural modeling of PI(4)P bound to the PIPBM previously suggested that the inositol 5-phosphate of PI(4,5)P_2_ would sterically clash with the γ-phosphate of ATP (18). However, when we measured the single molecule dwell times AF647-PIP5KB in TIRF-M imaging buffer containing either ADP, ATP, or nonhydrolyzable ATPγS, we found that the kinase could associate with PI(4,5)P_2_ membranes independent of the nucleotide chemistry (Fig. 3*B*). In fact, the presence of ATP caused AF647-PIP5KB to dwell slightly longer (τ_1_ = 0.463 ± 0.16 s) on membranes thano kinases visualized in the presence of ADP (τ_1_ = 0.365 ± 0.11 s) or ATPγS (τ_1_ = 0.329 ± 0.004 s). This suggests that nucleotide binding does not sterically occlude the interaction between PIP5K and PI(4,5)P_2_, which led us to hypothesize that the conserved PIPBM could potentially regulate PI(4,5)P_2_ lipid binding. To test whether the PIPBM is required for the PI(4,5)P_2_ lipid interactions, we introduced mutations that were homologous to zPIP5KA (*i.e.* K238 and R244) into human PIP5KB (*i.e.* K205A and R211A). We found that disrupting the PIPBM strongly diminished bulk membrane binding of PIP5K on PI(4,5)P_2_-containing membranes (Fig. 3*C*). Consistent with our bulk membrane localization experiment, single molecule characterization of AF647-PIP5KB (K205A/R211A) revealed a 2.4-fold decrease in the average dwell time compared to AF647-PIP5KB (Fig. 3*D*). While we did see greatly diminished dwell time, we did not observe a complete loss of PI(4,5)P_2_ binding. This shorter dwell time could be explained by the remaining specificity loop-binding interaction. To determine if our *in vitro* membrane-binding results are consistent with *in vivo* membrane-binding dynamics, we expressed mEos-PIP5KB (K205A, R211A) in HEK293T cells and quantified the single molecule plasma membrane dwell time (Fig. 3*E*). Consistent with the mutant having a reduced frictional drag coefficient in the plasma membrane due to fewer lipid interactions, the average single molecule displacement of mEos-PIP5KB (K205A/R211A) was significantly larger than mEos-PIP5KB (Fig. 3*F*). The average single molecule dwell time for mEos-PIP5KB (K205A/R211A) was also shorter than mEos-PIP5KB, reflecting a loss of molecular interactions (Fig. 3*G*). Fitting all the observed single molecule-binding events revealed that a larger fraction of mEos-PIP5KB (K205A/R211A, 34%) molecules displayed a transient membrane-binding behavior than mEos-PIP5KB (22%) (Fig. 3*H*). This data suggests that the PIPBM serves a role in regulating PIP5K plasma membrane localization in cells. However, other membrane localization mechanisms such as PIP5K dimerization (34, 36) and recruitment by PIP4K (38, 40) could mask the severity of the K205A/R211A localization phenotype observed *in vivo*.

Previous biochemical characterization of the zPIP5KA (R244A) mutant revealed that the PIPBM is critical for phosphorylating PI(4)P to generate PI(4,5)P_2_ (18). Our data also suggests that mutations in the PIPBM reduce PIP5K localization to membranes containing PI(4,5)P_2_. This led us to hypothesize that the previously observed loss in zPIP5KA kinase activity could be partially due to a disruption in membrane binding. This would be consistent with zPIP5KA (R244A) displaying no change in the ATP hydrolysis rate measured in solution (18). To test this hypothesis, we utilized the SpyTag-SpyCatcher interaction (41, 42) to rescue PIP5KB (K205A/R211A) membrane localization and measure lipid kinase activity (Fig. 3*I*). For these experiments, SpyCatcher was conjugated to a bilayer *via* a maleimide lipid. SpyTag-mNG-PIP5KB (K205A/R211A) was then flowed over the supported membrane in buffer lacking ATP and allowed to form an isopeptide bond with membrane-conjugated SpyCatcher. Unbound kinase in solution was washed out of the sample chamber before initiating the kinase reaction by adding ATP-containing buffer and the PI(4,5)P_2_ biosensor, Cy3-PLC*δ*. Consistent with the previous characterization of zPIP5KA (R244A), we found that nonmembrane-tethered SpyTag-PIP5KB (K205A/R211A) mutant was unable to catalyze PI(4,5)P_2_ production in solution (Fig. 3*J*). However, upon tethering to SpyTag-PIP5KB (K205A/R211A) to SLBs *via* SpyCatcher, we were able to localize and rescue PI(4,5)P_2_ production. Taken together, these data confirm that the site of high affinity binding and positive feedback come from binding PI(4,5)P_2_ lipids in the PIPBM. This high affinity binding interactions controls membrane localization of PIP5K both *in vitro* and *in vivo*.

### The PIPBM displays broad specificity for anionic lipids

**Figure 4:**
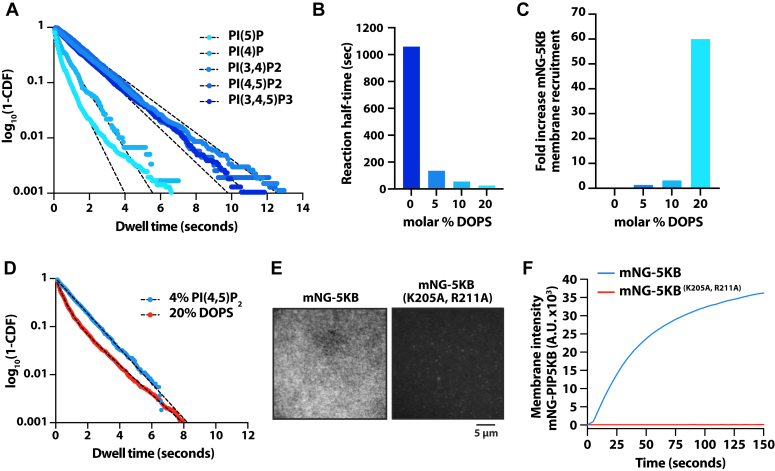
**The PIP5K PIPBMdisplaysbroad specificity for anionic lipids.***A*, single molecule dwell time distributions measured in the presence of mNG-PIP5KB on SLBs containing 4% of the indicated PIP lipid. *B*, kinetics of PIP5KB-dependent lipid phosphorylation measured in the presence of 0 to 20% DOPS. All membranes contain 2% PI(4)P and reactions were monitored using 20 nM Cy3-PLC*δ*. Kinetics were quantified by comparing the half-time for reaction completion. *C*, quantification of the fold increase of membrane recruitment measured in the presence of 15 nM mNG-PIP5KB on SLBs containing 0 to 20% DOPS. The fold increase in membrane binding we calculated relative to the membrane intensity of mNG-PIP5K measured in the absence of DOPS. *D*, single molecule dwell time analysis of mNG-PIP5KB in the presence of 4% PI(4,5)P_2_ or 20% DOPS. *E*, representative TIRF-M images showing the membrane localization of 15 nM mNG-PIP5K or mNG-PIP5K (Κ205A/R211A). Membrane composition: 20% DOPS, 80% DOPC. *F*, kinetics of bulk membrane recruitment of images shown in (*E*). See Table 1 for single molecule dwell times (τ_1_ and τ_2_) and statistics.

While the canonical activity of PIP5K is the phosphorylation of PI(4)P to produce PI(4,5)P_2_, PIP5K can also phosphorylate PI(3)P and PI(5)P, albeit less efficiently compared to PI(4)P (17, 18). In addition, PIP5K has also been shown to catalyze the synthesis of the important signaling lipid PI(3,4,5)P_3_ using PI(3,4)P_2_ as a substrate (18). Having established that the PIPBM regulates membrane interactions with PI(4,5)P_2_, we aimed to determine whether the PIPBM controls broad specificity for anionic lipids. For this, we created SLBs and performed single particle tracking of mNG-PIP5K in the presence of various types of PIP lipids (Fig. 4*A* and Table 1). We found that mNG-PIP5K bound singly phosphorylated PI(4)P and PI(5)P lipids with a similarly low affinity, while interactions with either doubly or triply phosphorylated PIP lipids (PI(3,4)P_2_, PI(4,5)P_2_, and PI(3,4,5)P_3_) displayed longer dwell times (Fig. 4*A* and Table 1). To test whether PIP5KB prefers to bind doubly and triply phosphorylated PIP lipids due to the structure of the inositol head group *versus* the total negative charge, we examined the membrane-binding properties of mNG-PIP5KB in the presence of PS lipids. We chose PS because its headgroup has a distinct chemical structure compared to PIP lipids but retains a negative charge. PS lipids have also previously been shown to activate PIP5K, though the molecular basis of this activation was not understood (43). In agreement with published data, we found that increasing the molar percentage of PS enhanced PIP5K activity, with a physiologically relevant concentrations of 20% PS lipids increasing the kinase activity 42.5-fold compared to membranes that contained 2% PI(4)P and no PS lipids (Fig. 4*B*). Correlated with the observed increase in kinase activity, mNG-PIP5KB localization displayed a nonlinear response to PS membrane density. At the physiologically relevant concentration of 20% PS, mNG-PIP5KB membrane localization increased 60-fold compared to membranes lacking PS lipids (Fig. 4*C*). Next, we compared the single molecule dwell times of mNG-PIP5KB associated with supported membranes containing either 4% PI(4,5)P_2_ or 20% PS lipids. In the presence of either of these lipid species, mNG-PIP5KB displayed similarly long membrane dwell times (Fig. 4*D* and Table 1). To determine if the interaction with PS lipids was mediated by the PIPBM, we measured the bulk membrane localization of mNG-PIP5K (K205A, R211A). Like our characterization of mNG-PIP5K (K205A, R211A) membrane binding in the presence of PI(4,5)P_2_ (Fig. 3*C*), the PIPBM mutant was unable to localize to membranes containing 20% PS lipids (Fig. 4, *E* and *F*). Taken together, these data demonstrate that PIP5K is localized to PS through association of the lipid with the PIPBM, supporting a model in which the PIPBM functions as a broad specificity anionic lipid sensor.

**Table 1:** 

Protein visualized	Membrane composition	*τ*_*1*_*± SD (sec)*	*τ*_*2*_*± SD (sec)*	*α ± SD*	*N*	*n*
mNG-PIP5KB	4% PI(4)P	0.469 ± 0.22	—	—	3	6251
mNG-PIP5KB	4% PI(5)P	0.557 ± 0.14	—	—	3	21,425
mNG-PIP5KB	4% PI(3,4)P_2_	0.854 ± 0.32	—	—	3	5461
mNG-PIP5KB	4% PI(4,5)P_2_	1.05 ± 0.13	—	—	3	3993
mNG-PIP5KB	4% PI(3,4,5)P_3_	0.503 ± 0.07	1.419 ± 0.028	0.42 ± 0.085	3	16,930
mNG-PIP5KB	20% PS	0.322 ± 0.19	1.414 ± 0.49	0.74 ± 0.23	3	11,351
mNG-PIP5KB	2% PI(4,5)P_2_	0.468 ± 0.019	—	—	3	7075
mNG-PIP5KB (K138A)	2% PI(4,5)P_2_	0.285 ± 0.004	—	—	3	7423
mNG-PIP5KB (D266K)	2% PI(4,5)P_2_	0.619 ± 0.071	—	—	3	4667
mNG-PIP5KB (D350A)	2% PI(4,5)P_2_	0.228 ± 0.004	—	—	3	3546

SD = standard deviation from the indicated number of technical replicates.

*N* = # of SLBs or cells used for calculating the mean dwell times (*i.e.* technical replicates).

n = total number of molecules tracked across all of the indicated number of technical replicates (*N*).

alpha (α) = fraction of molecules with characteristic dwell time (τ_1_).

Membrane composition equals DOPC plus the indicated molar percentage of PIP or PS lipids.

### Deciphering the relationship between PIP5K membrane recruitment and catalysis

**Figure 5:**
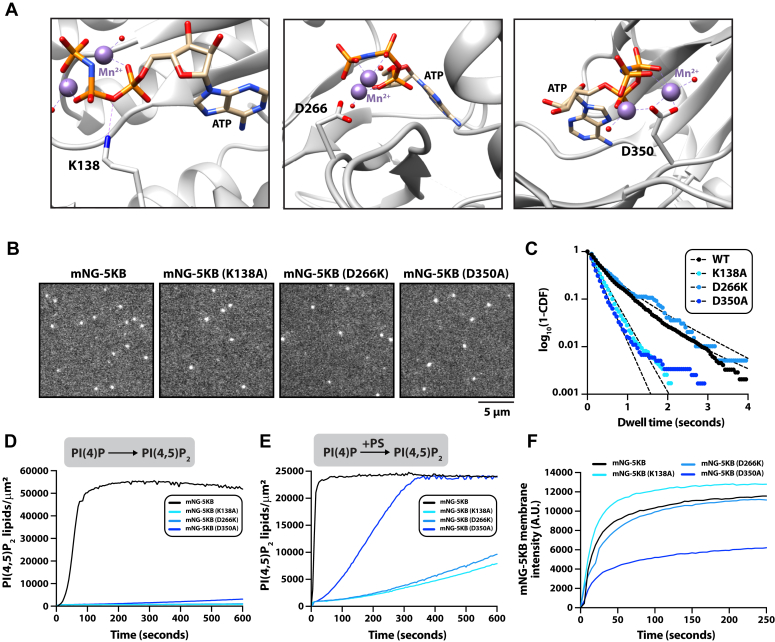
**Deciphering the relationship between PIP5K catalysis and membrane recruitment.***A*, structure of zPIP5KA (5E3U.pdb) showing the position of residues that are important for catalysis. Residue numbers represent human PIP5KB amino acids that are homologous to the zPIP5KA residues shown in the structure. *B*, representative TIRF-M images showing the localization of mNG-PIP5KB (WT and mutants). *C*, single molecule dwell time distributions for mNG-PIP5KB (WT and mutants) measured. See Table 1 for single molecule dwell times (τ_1_) and statistics. *B* and *C*, membrane composition: 98% DOPC, 2% PI(4,5)P_2_. *D* and *E*, lipid kinase activity measured using TIRF-M in the presence of 10 nM PIP5KB (WT and mutants). Production of PI(4,5)P_2_ was monitored in the presence of 20 nM Cy3-PLC*δ*. *D*, membrane composition: 96% DOPC, 4% PI(4)P. *E*, membrane composition: 78% DOPC, 2% PI(4)P, 20% DOPS. *F*, bulk membrane recruitment measured by TIRF-M in the presence of 20 nM mNG-PIP5KB (WT and mutants). Membrane composition: 98% DOPC, 2% PI(4,5)P_2_.

Our membrane-binding studies of the PIPBM mutant revealed that residues that have previously been shown to be critical for lipid kinase activity also regulate PIP5K membrane localization. This raised questions about whether other commonly studied “kinase dead” mutants are defective in membrane binding. For this reason, we aimed to identify a PIP5K “kinase dead” mutant that lacks lipid kinase activity but retains the membrane-binding dynamics characteristic of the WT enzyme. To validate a separation of function mutant, we rationalized that PIP5K must have a functional specificity loop and PIPBM. Looking at existing structural biochemistry data of zPIP5KA (18), we found that residue D350 helps coordinate a Mg^2+^/Mn^2+^ ions in the active site in PIP5KB (Fig. 5*A*). Mutations in this residue are predicted to destabilize the transition state following nucleophilic attack of the ATP gamma phosphate. In addition, residues K138 and D266 of PIP5KB serve important roles in stabilizing ATP in the active site. Using the mNG-PIP5K single molecule TIRF assay, we quantified the membrane-binding properties of fluorescently labeled PIP5KB (K138A, D266A, and D350A) on supported membrane-containing 2% PI(4,5)P_2_ (Fig. 5*B*). We found that only the D266K mutant exhibits membrane-binding properties similar to WT PIP5KB (Fig. 5*C*). This was observed when we measured the single molecule dwell times (Fig. 5*C* and Table 1) and bulk membrane recruitment (Fig. 5*D*). Next, we measured the catalytic efficiency of each mutant on supported membranes containing an initial concentration of 4% PI(4)P and no additional anionic lipids. Under these conditions, none of the mutants displayed significant levels of activity compared to WT PIP5KB (Fig. 5*E*). When we repeated these experiments on supported membranes containing 20% PS lipids, we were able to stimulate the activity of PIP5KB (D350A) through enhanced membrane localization (Fig. 5*F*). However, the K138A and D266K mutants still displayed weak activity even though their membrane localization dynamics were similar to WT PIP5KB. Together, these results indicate that some PIP5K “kinase dead” mutants display reduced catalytic efficiency due to the inability to bind to anionic lipids, which controls membrane localization.

## Discussion

Previous structural biochemistry studies have implicated both the specificity loop and PIPBM (also known as the substrate-binding motif) of PIP5K in regulating substrate specificity and kinase activity (16, 17, 18, 35). These molecular details contribute to two potential models that describe PIP5K membrane docking and catalysis. In a single binding pocket model, the specificity loop adopts a helical conformation positioned near the PIPBM. These two motifs function in concert to form a single unified PIP lipid-binding site responsible for substrate specificity. This model is supported by mutational analysis and chemical crosslinking studies that show the specificity loop and PIPBM move into close proximity of each other following substrate binding (18). Alternatively, in a two-step binding model, the specificity loop regulates the initial membrane sensing and docking step, while the substrate binding and PI(4)P phosphorylation is mediated by the PIPBM. In this model, the specificity loop holds the PIP5K enzyme at the membrane until the PI(4)P lipid interaction is repositioned to interact with the PIPBM. This model is supported by molecular dynamics simulations showing that PI(4)P binds to the specificity loop prior to engaging the PIPBM (37). Through the direct visualization of fluorescently labeled PIP5K on supported lipid bilayers using TIRF microscopy, we found that mutating either the specificity loop or PIPBM disrupts PIP5K lipid kinase activity, membrane localization, and positive feedback. In support of the two-step membrane recruitment mechanism, we find that mutating the specificity loop reduces the membrane-binding frequency (*k*_*ON*_) but only moderately affects the dissociation rate constant (*k*_*OFF*_) of PIP5K. The ability of the PIPBM to facilitate membrane association in the absence of a functional specificity loop provides support for a two-step membrane recruitment mechanism. Although membrane recruitment of PIP5K is more robust in the presence of both a functional specificity loop and PIPBM, each motif can individually contribute to membrane binding.

Previous research has established the specificity loop as the determinative motif that controls substrate specificity of the type I and II PIP kinases, PIP4K and PIP5K (16, 17, 18, 35). Studies have identified specific residues in the PIP4K and PIP5K specificity loops that can be swapped to switch substrate recognition and modulate catalysis (17, 18). The structural basis of sequence-dependent changes in lipid specificity PIP4K and PIP5K, however, remains unclear. Our characterization of chimeric fusion proteins revealed that the PIP5K specificity loop can confer specificity for either PI(4)P and PI(4,5)P_2_ when attached to another peripheral membrane-binding protein. Fusing the PIP5KB specificity loop to the PH domain of PLCδ enhanced the ability of the mNG-(5K-SL)-PLC*δ* chimeric protein to interact with PI(4)P and PI(4,5)P_2_ lipids, but not PI(5)P (Fig. 2). These findings support a model in which the specificity loop has preferred PIP lipid interactions, rather than functioning as a broad lipid membrane sensor.

The PIPBM has previously been described as the active site of the type I, II, and III PIP lipid kinases (18). The finding that the PIPBM also strongly binds PI(4,5)P_2_ lipids raises questions concerning how PIP5K can exhibit positive feedback based on product binding when PI(4,5)P_2_ appears to function as competitive inhibitor. If PI(4,5)P_2_ binds to the “active site,” we expect to observe attenuated lipid kinase activity following PI(4)P phosphorylation. One mechanism that could resolve this paradox is through membrane-mediated dimerization of PIP5K. We previously reported that PIP5K dimerizes in a density-dependent manner and that dimerization potentiates lipid kinase activity through a mechanism consistent with allosteric regulation (34). Molecular dynamics simulations have shown that only a single kinase domain of a PIP5K dimer is able to associate with membrane lipids (37). Quantitative analysis of monomeric and dimeric mNG-PIP5K membrane surface densities also support a model in which only a single kinase domain can engage PI(4,5)P_2_ on a membrane (34). This may allow dimeric PIP5K to bypass PI(4,5)P_2_-dependent inhibition by toggling membrane engagement of kinase domains between cycles of catalysis. Additional biochemical and molecular dynamics simulations are required to understand the mechanism for bypassing competitive inhibition due to PI(4,5)P_2_ binding.

Catalytically dead PIP5K mutants are often used in cellular PIP5K studies (38, 44, 45, 46). Many studies, however, used mutations to the PIPBM to achieve their “catalytically dead” PIP5K mutants. This is unideal, as we show in this study that mutating the PIPBM leads perturbs membrane localization. We sought to identify candidate catalytically dead PIP5K mutants for the field to use in future *in vivo* studies. We used single molecule TIRF microscopy to characterize several catalytically dead PIP5K mutants (*i.e.* K138A, D266K, and D350A) with mutations outside of the PIPBM that have previously been used in a variety of cell biological studies (38, 44, 45, 46, 47, 48, 49, 50, 51, 52, 53, 54). We found that PIP5KB (D266K) was the ideal separation of function mutant because it displayed single molecule membrane-binding properties that were nearly identical to the WT kinase while showing no discernable activity on PI(4)P membranes and greatly reduced activity in the presence of a physiologically relevant concentrations of PS lipids. It is important to note that in the presence of 20% phosphatidylserine, none of the “catalytically dead” PIP5K mutants were fully inactive. Considering that the cellular environment contains a variety of antagonists, including PIP lipid phosphatases and PIP4K, the basal level of activity displayed by the PIP5KB (D266K) mutant will likely be suppressed *in vivo*.

Previous studies have shown that PIP5K enzymes target the inner leaflet of the cellular plasma membrane (24, 38). The prevailing hypothesis in the field is that PIP5K targets the membrane through direct association with the lipid substrate, PI(4)P, and interactions with peripheral membrane-binding proteins (14, 25, 36, 55, 56). However, if PIP5K preferentially binds to its substrate lipid, PI(4)P, why does it target the inner leaflet of the plasma membrane and not other PI(4)P-containing intracellular membranes? Previous characterization by immunofluorescence has shown that a large abundance of cellular PI(4)P exists at the Golgi cisternae (57, 58, 59). The overall lack of strong PIP5K localization to the Golgi suggests that its substrate, PI(4)P, is not the major lipid interaction that promotes plasma membrane localization of PIP5K. Our data provides a different explanation for preferential inner leaflet targeting. First, we show that PIP5K binds PI(4,5)P_2_ lipids with higher affinity than PI(4)P lipids (Fig. 4*A*), consistent with previous findings (33, 34). Previous work, however, demonstrated that disrupting PI(4,5)P_2_ lipid concentrations diminishes but does not fully abolish plasma membrane localization of PIP5K (24). Our study explains this result by establishing that PIP5K recruits to membranes containing high molar concentrations of anionic lipids like PS. However, unlike PI(4,5)P_2_ binding, which occurs even at low molar percentages, PS must be present at high molar concentrations >15% before we detect robust membrane recruitment of PIP5K (Fig. 4*C*). This switch-like recruitment to PS provides a possible explanation for PIP5K inner leaflet targeting. In mammalian cell membranes, despite PS being present at all intracellular membranes, it is only highly abundant (>15%) in the inner leaflet of the plasma membrane (60, 61).

Researchers recently discovered that PIP4K negatively regulates PIP5K activity, which establishes a homeostatic mechanism for maintaining stable PI(4,5)P_2_ levels at the plasma membrane (38, 40). Plasma membrane recruitment of PIP4K requires elevated levels of PI(4,5)P_2_, where it then attenuates PIP5K catalytic activity through an unknown mechanism (38). *In vitro* experiments confirmed that PIP4K requires a higher molar concentration of PI(4,5)P_2_ lipids for membrane localization compared to PIP5K (38). Why PIP4K had a reduced sensitivity to PI(4,5)P_2_ lipids compared to PIP5K was unclear. Here, we report that the difference in PI(4,5)P_2_ lipid sensitivity can be attributed to the divergence in the PIP4K and PIP5K specificity loop sequences. Swapping specificity loops enhanced the membrane-binding frequency of PIP4K, even on SLBs containing 1% PI(4,5)P_2_. Overall, our results show that the PIP4K and PIP5K specificity loop is a major factor that controls sensitivity to PI(4,5)P_2_ lipids. These findings reveal intricate mechanisms underlying membrane targeting and lipid kinase activity within the phosphoinositide signaling pathway, offering insights into potential avenues for modulating cellular PI(4,5)P_2_ levels by PIP4K and PIP5K during cell signaling.

## Experimental procedures

### Molecular biology

The gene coding the PH domain derived from human phospholipase C-*δ*1 (PLCδ Accession #P51178.2), human phosphatidylinositol 4-phosphate 5-kinase type-1 beta (hPIP5KB; Uniprot #O14986), and human phosphatidylinositol 5-phosphate 4-kinase type-2 beta (hPIP4KB; Uniprot #P78356) were derived from codon-optimized genes synthesized by GeneArt (Invitrogen). The gene encoding mEos3.2 (62) was PCR-amplified and cloned in-frame with PIP5KB to create a N-terminal fusion for mammalian cell expression. Gene sequences were subcloned into either bacterial, insect cell, or mammalian expression vectors using Gibson assembly (63). Plasmids containing PIP5KB mutations (*i.e.* W365A, W365P, D51R, K205A, R211A, K138A, D350A, D266K, *etc.*) were generated through site-directed mutagenesis using the PfuUltra High-Fidelity DNA polymerase (Agilent, cat# 600380). Chimeric mNG-PIP5K, mNG-PLCδ, and mNG-Specificity Loop-PLCδ were created using PCR amplification of genes *via* AccuPrime *Pfx* Master Mix (Thermo Fisher Scientific, Cat#12344040) then combined with a digested plasmid using Gibson Assembly. The complete ORF of all vectors used in this study were sequenced by Azenta (formerly Genewiz) and Plasmidsaurus (University of Oregon) to ensure constructs lacked deleterious mutations. Each protein expression construct was screened for optimal yield and solubility in either bacteria (BL21 DE3 Star, Rosetta, *etc.*) or *Spodoptera frugiperda* (Sf9) insect cells. See Supplementary file for the amino acid sequences used during recombinant protein expression or transient transfection in HEK293T cells. Note that there have previously been inconsistencies in nomenclature between human and mouse PIP5K paralogs. In this manuscript, PIP5KB refers to the human PIP5KB paralog. All plasmids created and used within this manuscript function under this human nomenclature.

### Protein purification

#### PIP5KB and mNG-PIP5K

WT and mutant PIP5K proteins were recombinantly expressed and purified as previously described (33, 34). Gene sequences encoding PIP5KB and mutants (*i.e.* W365P, D51R, and K205A/R211A) were cloned into FastBac1 vectors in frame with an N-terminal his_6_-MBP-TEV-GGGGG or his_6_-TEV-mNG-GGGGG and expressed under the polyhedrin (pH) promoter. BACMIDS and baculoviruses were generated as previously described (33). For large protein expression, high five cells were infected with baculovirus using an optimized multiplicity of infection, typically 2% vol/vol. Infected insect cells were grown for 48 h at 27 °C in ESF 921 serum-free insect cell culture medium (Expression Systems, Cat# 96-001-01). Cells were then harvested by centrifugation, washed with 1× PBS [pH 7.2], resuspended in cell storage buffer (1× PBS [pH 7.2], 10% glycerol, 2× Sigma protease inhibitor table), and then stored in the −80 °C freezer. For purification, frozen insect cell pellets harvested from 2 to 4 L of liquid cell culture were thawed at room temperature in a water bath and lysed into buffer containing 50 mM Na_2_HPO_4_ [pH 8.0], 10 mM imidazole, 400 mM NaCl, 1 mM PMSF (added twice, once before homogenization and once after), 5 mM beta-mercaptoethanol (BME), 100 μg/ml DNase, SIGMAFAST protease inhibitor cocktail tablets, EDTA-free (Sigma, Cat# S8830-20TAB) per 100 ml lysis buffer. Cells in this buffer were lysed using a dounce homogenizer. Lysate was clarified by centrifugation at 36,000 rpm (140,000*g*) for 60 min under vacuum using a Beckman Ti-45 rotor at 4 °C. Lysate was then batch bound to 5 ml of Ni-NTA Agarose (Qiagen, Cat# 30230) resin at 4 °C for 2 h in a beaker set on a stir plate. Resin was then collected in 50 ml tubes, centrifuged, and washed with buffer containing 50 mM Na_2_HPO_4_ [pH 8.0], 10 mM imidazole, 400 mM NaCl, and 5 mM BME and centrifuged again before being transferred to gravity flow column in more wash buffer. Ni-NTA resin with his_6_-MBP-TEV-GGGGG-PIP5K bound was then eluted into buffer containing 500 mM imidazole. Peak fractions were pooled, combined with 200 μg/ml his6-TEV(S291V) protease, and dialyzed against 4 L of buffer containing 20 mM Tris [pH 8.0], 200 mM NaCl, 2.5 mM BME for 16 to 18 h at 4 °C. Dialysate was then combined 1:1 with 20 mM Tris [pH 8.0], 1 mM TCEP (∼100 mM NaCl final). Precipitation was removed by centrifugation and 0.22 μm syringe filtration. Clarified dialysate was then bound to a MonoS cation exchange column (GE Healthcare, Cat# 17-5168-01) equilibrated in 20 mM Tris [pH 8.0], 100 mM NaCl, 1 mM TCEP buffer. Proteins were resolved over a 10 to 100% linear gradient (0.1–1 M NaCl, 45 CV, 45 ml total, 1 ml/min flow rate). PIP5K homologs and paralogs typically eluted from the MonoS column in the presence of 370 to 450 mM NaCl. Peak fractions containing PIP5K were pooled, concentrated in a 30 kDa MWCO Vivaspin 6 centrifuge tube (GE Healthcare, Cat# 28-9323-17), and loaded onto a 24 ml Superdex 200 10/300 Gl (GE Healthcare, Cat# 17-5174-01) size-exclusion column equilibrated in 20 mM Tris [pH 8.0], 200 mM NaCl, 10% glycerol, 1 mM TCEP. Peak fractions were concentrated in a 30 kDa MWCO Vivaspin 6 centrifuge tube and snap frozen at a final concentration of 10 to 40 μM using liquid nitrogen. GGGGG-PIP5KB (WT and mutants) were labeled with Alexa647-LPETGG using sortase-mediated peptide ligation as previously described (33).

#### PIP4K2B and PIP4K2B (5K-SL)

Codon-optimized gene sequence encoding human PIP4K2B isoform 2 (Uniprot #P78356) was cloned into a pETM-derived bacterial expression vector to create the following fusion protein: his_6_-SUMO3-GGGGG-PIP4K2B (1–416aa). Recombinant PIP4K2B was expressed in BL21(DE3) Star *Escherichia coli* as previously described (38). Using 2 to 4 L of terrific broth, bacterial cultures were grown at 37 °C until A_600_ = 0.6. Cultures were then shifted to 18 °C for 1 h to cool down. Protein expression was induced with 50 μM IPTG and bacteria-expressed protein for 20 h at 18 °C before being harvested by centrifugation. For purification, cells were lysed into buffer containing 50 mM Na_2_HPO_4_ [pH 8.0], 400 mM NaCl, 0.4 mM BME, 1 mM PMSF (add twice, 15 min intervals), DNase, 1 mg/ml lysozyme using a microtip sonicator. Lysate was centrifuged at 16,000 rpm (35,172*g*) for 60 min in a Beckman JA-17 rotor chilled to 4 °C. Lysate was circulated over 5 ml HiTrap Chelating column (GE Healthcare, Cat# 17-0409-01) that had been equilibrated with 100 mM CoCl_2_ for 1 h, washed with MilliQ water, and followed by buffer containing 50 mM Na_2_HPO_4_ [pH 8.0], 400 mM NaCl, 0.4 mM BME. Recombinant PIP4K2B was eluted with a linear gradient of imidazole (0–500 mM, 8 CV, 40 ml total, 2 ml/min flow rate). Peak fractions were pooled, combined with 50 μg/ml of his6-SenP2 (SUMO protease), and dialyzed against 4 L of buffer containing 25 mM Na_2_HPO_4_ [pH 8.0], 400 mM NaCl, and 0.4 mM BME for 16 to 18 h at 4 °C. Following overnight cleavage of the SUMO3 tag, dialysate containing his6-SUMO3, his6-SenP2, and GGGGG-PIP4K2B was recirculated for at least 1 h over a 5 ml HiTrap(Co^+2^) chelating column. Flow-through containing GGGGG-PIP4K2B was then concentrated in a 30 kDa MWCO Vivaspin 6 before loading onto a Superdex 200 size-exclusion column equilibrated in 20 mM Hepes [pH 7], 200 mM NaCl, 10% glycerol, 1 mM TCEP. In some cases, cation exchange chromatography was used to increase the purity of GGGGG-PIP4K2B before resolving on the Superdex 200 column. In those cases, we equilibrated a MonoS column 20 mM Hepes [pH 7], 100 mM NaCl, 1 mM TCEP buffer. PIP4K2B (pI = 6.9) bound to the MonoS was resolved over a 10 to 100% linear gradient (0.1–1 M NaCl, 30 CV, 30 ml total, 1.5 ml/min flow rate). Peak fractions collected from the Superdex 200 were concentrated in a 30 kDa MWCO Vivaspin 6 centrifuge tube and snap frozen at a final concentration of 20 to 80 μM using liquid nitrogen.

Codon-optimized gene sequence encoding human PIP4K2B isoform 2 (Uniprot #P78356) was modified by PCR-based insertion to swap the PIP4K2B specificity loop (372–384aa; DTKKKAAHAAKTVKHGAGAEI) for the PIP5KB specificity loop (353–373aa; RLMKKLEHSWKALVYDGDTV). This produced a chimeric PIP4K2B enzyme with a PIP5K specificity loop, which is referred to as PIP4K2B (5K-SL). The his_6_-SUMO3-GGGGG-PIP4K2B (5K-SL) protein was purified as described above for WT PIP4K2B. Both PIP4K2B and PIP4K2B (5K-SL) were labeled on an N-terminal GGGGG motif with Cy5-LPETGG using sortase-mediated peptide ligation (33, 34, 64). Following labeling, Cy5-PIP4KB and Cy5-PIP4KB (5K-SL) were resolved on a Superdex 200 size-exclusion column equilibrated in 20 mM Hepes [pH 7], 200 mM NaCl, 10% glycerol, 1 mM TCEP. This removed free Cy5-LPETGG, Sortase (16 kDa), and a small amount of aggregated Cy5-PIP4KB. Peak fractions were pooled from the Superdex 200 column and concentrated using a 30 kDa MWCO Vivaspin 6 centrifuge tube before being snap frozen in liquid nitrogen at a final concentration of 10 to 20 μM.

#### PLCδ-PH domain

This protein was expressed and purified as previously described (33). Briefly, human PLCδ-PH domain (11–140aa) was expressed in BL21 (DE3) Star *E. coli* as a his_6_-SUMO3-(Gly)_5_-PLCδ (11–140aa) fusion protein. Following growth at 37 °C in terrific broth to an A_600_ of 0.8, cultures were shifted to 18 °C for 1 h, induced with 0.1 mM IPTG, and allowed to express protein for 20 h at 18 °C before being harvested. Cells were lysed into 50 mM Na_2_HPO_4_ [pH 8.0], 300 mM NaCl, 0.4 mM BME, 1 mM PMSF, 100 μg/ml DNase using a microfluidizer. Lysate was then centrifuged at 16,000 rpm (35,172*g*) for 60 min in a Beckman JA-17 rotor chilled to 4 °C. Lysate was circulated over 5 ml HiTrap chelating column (GE Healthcare, Cat# 17-0409-01) charged with 100 mM CoCl_2_ for 1 h. Bound protein was then eluted with a linear gradient of imidazole (0–500 mM, 8 CV, 40 ml total, 2 ml/min flow rate). Peak fractions were pooled, combined with SUMO protease (50 μg/ml final concentration), and dialyzed against 4 L of buffer containing 50 mM Na_2_HPO_4_ [pH 8.0], 300 mM NaCl, and 0.4 mM BME for 16 to 18 h at 4 °C. Dialysate containing SUMO-cleaved protein was recirculated for 1 h over a 5 ml HiTrap chelating column. Flow-through containing (Gly)_5_-PLCδ (11–140aa) was then concentrated in a 5 kDa MWCO Vivaspin 20 before being loaded on a Superdex 75 size-exclusion column equilibrated in 20 mM Tris [pH 8.0], 200 mM NaCl, 10% glycerol, 1 mM TCEP. Peak fractions containing (Gly)_5_-PLCδ (11–140aa) were pooled and concentrated to a maximum concentration of 75 μM (1.2 mg/ml) before snap freezing with liquid nitrogen and storage at −80 °C. As previously described, GGGGG-PLCδ (11–140aa) was labeled with either AF488 or Cy3 using sortase-mediated peptide ligation (33, 34).

#### mNG-PLCδ-PH and mNG-(5K-SL)-PLCδ-PH

Genes encoding chimeric mNeonGreen-hPLCδ-PH domain (11–140aa) and mNeonGreen-hPIP5K Specificity Loop (353–373aa)-hPLCδ-PH domain (11–140aa) were cloned into bacterial expression vectors. Each construct was expressed in BL21 (DE3) Star *E. coli* as his_10_-TEV fusion proteins. Bacteria were grown at 37 °C in terrific broth to an A_600_ of 0.8. Cultures were induced with 0.1 mM IPTG and allowed to express protein for 20 h at 18 °C before being harvested. Cells were lysed into 50 mM Na_2_HPO_4_ [pH 8.0], 400 mM NaCl, 0.5 mM BME, 1 mM PMSF, 100 μg/ml DNase using tip sonication (45% amplitude, 5 s on, 10 s off). Lysate was then centrifuged at 16,000 rpm (35,172*g*) for 60 min in a Beckman JA-17 rotor chilled to 4 °C. Lysate was circulated over 5 ml HiTrap chelating column (GE Healthcare, Cat# 17-0409-01) charged with 100 mM CoCl_2_ for 1 h. Bound protein was then eluted with a linear gradient of imidazole (0–500 mM, 8 CV, 40 ml total, 2 ml/min flow rate). Peak fractions were pooled and combined with 200 μg/ml his6-TEV(S291V) protease and dialyzed against 4 L of buffer containing 50 mM Na_2_HPO_4_ [pH 8.0], 400 mM NaCl, and 0.4 mM BME for 16 to 18 h at 4 °C. Dialysate containing cleaved His_10_-TEV protein was recirculated for 1 h over a 5 ml HiTrap chelating column. Flow-through containing mNG-PLCδ or mNG-(5K-SL)-PLCδ were then concentrated in a 50 kDa MWCO Vivaspin 20 before being loaded on a Superdex 75 size-exclusion column equilibrated in 25 mM Tris [pH 8.0], 150 mM NaCl, 10% glycerol, 1 mM TCEP. Peak fractions containing either mNG-PLCδ or mNG-(5K-SL)-PLCδ were pooled and concentrated to 4 to 20 μM before snap freezing with liquid nitrogen and storage at −80 °C.

#### Preparation of small unilamellar vesicles

The following lipids were used to generated small unilamellar vesicles (SUVs): 1,2-dioleoyl-sn-glycero-3-phosphocholine (18:1 DOPC, Avanti # 850375C), L-α-phosphatidylinositol-4-phosphate (Brain PI(4)P, Avanti Cat# 840045X), 1,2-dioleoyl-sn-glycero-3-phospho-(1′-myo-inositol-5′-phosphate) (PI(5)P, Avanti Cat# 850152P), L-α-phosphatidylinositol-4,5-bisphosphate (Brain PI(4,5)P_2_, Avanti Cat# 840046X), D-myo-phosphatidylinositol 3,4,5-trisphosphate (PI(3,4,5)P_3_ diC16, Echelon Cat# P-3916-100μg), D-myo-phosphatidylinositol 3,4-bisphosphate (PI(3,4)P_2_ diC16, Echelon Cat# P-3416-100μg), 1,2-dioleoyl-*sn-*glycero-3-phospho-L-serine (18:1 DOPS, Avanti Cat# 840035C), 1,2-dioleoyl-sn-glycero-3-phosphoethanolamine-N-[4-(p-maleimidomethyl)cyclohexane-carboxamide] (18:1 MCC-PE, Avanti Cat# 780201C). To make liposomes, 2 μmoles of total lipids are combined in a 35 ml glass round bottom flask with 2 ml of chloroform. Lipids were dried to a thin film using rotary evaporation with the glass round-bottom flask submerged in a 42 °C water bath. The lipid film was then resuspended in 2 ml of PBS [pH 7.2], getting a final concentration of 1 mM total lipids. All lipid mixtures expressed as percentages (*e.g.* 98% DOPC, 2% PI(4)P) are equivalent to molar fractions. To generate SUVs, 1 mM total lipid mixture was extruded through a 0.05 μm pore size 19 mm polycarbonate membrane (Avanti, Cat# 610002) with filter supports (Avanti, Cat# 610014) on both sides of the polycarbonate membrane. Extruding hydrated lipids a total of 11 times achieved the desired SUV diameter of ∼50 nm.

#### Preparation of supported lipid bilayers

SLBs are formed on 25 x 75 mm coverglass (IBIDI, Cat# 10812). Coverglass first is cleaned with 2% Hellmanex III (Thermo Fisher Scientific, Cat# 14-385-864) heated to 60-70 °C in a glass coplin jar. This was incubated for at least 30 min. Once incubated, the coverglass was washed thoroughly with MilliQ water. Once cleaned, the coverglass was then etched in Piranha solution (1:3, hydrogen peroxide:sulfuric acid) for 10 to 15 min the same day SLBs were formed. Once etched, coverglass was again thoroughly rinsed with MilliQ water before being rapidly dried with nitrogen glass. Once dried, glass was adhered to a 6-well sticky-side chamber (IBIDI, Cat# 80608). SLBs were formed by flowing 30 nm SUVs diluted in PBS [pH 7.2] to a total lipid concentration of 0.25 mM and incubated for 30 min. IBIDI chambers were then washed with 5 ml of PBS [pH 7.2] to remove nonabsorbed SUVs. Membrane defects are blocked for 15 min with a 1 mg/ml beta casein (Thermo Fisher Scientific, Cat# 37528) diluted in 1× PBS [pH 7.4]. Before use as a blocking protein, frozen 10 mg/ml beta casein stocks were thawed, centrifuged for 30 min at 21,370*g*, and 0.22 μm syringe filtered. After blocking SLBs with beta casein, membranes were washed again with 1 ml of PBS, followed by 1 ml of kinase buffer before TIRF-M.

#### Membrane conjugation of SpyCatcher

Following beta-casein blocking of SLBs, membranes that contained MCC-PE lipids were washed into 1× PBS [pH 7.2] containing 0.1 mM TCEP. MCC-PE lipids were used to covalently couple SpyCatcher protein onto SLBs. For these SLBs, 100 μl of 30 μM SpyCatcher diluted in a 1× PBS [pH 7.2] and 0.1 mM TCEP buffer was added to the IBIDI chamber and incubated for 2 h at 23 °C. Once the coupling period passed, SLBs with MCC-PE lipids were then washed with 2 ml of 1× PBS [pH 7.2] containing 5 mM BME and incubated in this buffer for 15 min to quench the unreacted maleimide headgroups. SLBs were then washed with 2 ml of 1× PBS to remove unbound protein. Membranes were stored for up to 2 h in 1× PBS before being washed into imaging buffer to initiate TIRF-M measurements.

#### Assay for measuring the kinetics of PI(4,5)P_2_ production

The kinetics of PI(4)P phosphorylation were measured on SLBs formed in IBIDI chambers and visualized using TIRF microscopy as previously described (33, 34). Imaging or reaction buffer contained 20 mM Hepes [pH 7.0], 150 mM NaCl, 1 mM ATP, 5 mM MgCl_2_, 0.5 mM EGTA, 20 mM glucose, 200 μg/ml beta casein (Thermo Fisher Scientific, Cat# 37528), 20 mM BME, 320 μg/ml glucose oxidase (Serva, Cat# 22780.01 *Aspergillus niger*), 50 μg/ml catalase (Sigma, Cat# C40-100 MG Bovine Liver), and 2 mM Trolox (UV treated as previously described by Hansen *et al.* 2019). Perishable reagents (*i.e.* glucose oxidase, catalase, and Trolox) were added 5 to 10 min before image acquisition. For all experiments, we monitored the change in PI(4)P or PI(4,5)P_2_ membrane density using a solution concentration of 20 nM AF488-GGGGG-PLCδ or Cy3-GGGGG-PLCδ. Density of PIP lipids (lipids/μm^2^) was calculated assuming a footprint of 0.72 nm^2^ for DOPC lipids (33, 65).

#### Cell culture and live cell imaging

(HEK) 293T cells (ATCC, #CRL-3216) were cultured in DMEM + GlutMAX + High Glucose (4.5 g/L) + sodium pyruvate (110 mg/L) (Life Technologies, cat #10569010) supplemented with 10% FBS (Sigma, cat# F4135-500 Ml), penicillin (100 units/ml), and streptomycin (100 μg/ml). This cell line was acquired from the Barker Hall tissue culture facility at the University of California at Berkeley. Cells were grown in 10 cm dishes in humidified incubators at 37 °C in the presence of 5% CO_2_ and split at a confluency of 80 to 90% every 2 to 3 days. HEK293T cells were split using using 1.5 ml of 0.25% Trypsin. Trypsin was that quenched with 8.5 ml complete DMEM media containing 10% FBS. Cells were diluted 1:10 and seeded on a new 10 cm dish containing a total volume of 10 ml complete DMEM media warmed to 37 °C.

HEK293T cells were prepared for imaging by seeding cells at a confluency of 30% in an 18-well glass bottom chamber (IBIDI, Cat# 81817) 48-h before imaging. Cells were transfected 24-h before imaging with 0.1 μg of plasmid DNA using Lipofectamine 2000 (Thermo Fisher Scientific, Cat# 11668027). Prior to imaging cells, the complete DMEM media containing phenol red was aspirated from the chamber and replaced with 1× HBSS (20 mM HEPES [pH 7.2], 150 mM NaCl, 4 mM KCl, 1 mM MgCl_2_, 10 mM glucose). The chamber was mounted on an inverted Nikon Ti2 microscope with an OKO Touch heated stage. Cells were imaged through a 100× Nikon objective (1.49 NA) oil immersion TIRF objective with a heated flexible collar with temperature control. The correction collar on the 100× objective was adjusted for 37 °C imaging.

Single molecule imaging of PIP5KB in HEK293T utilized an N-terminal fusion of monomeric Eos3.2 (62), referred to as mEos throughout the manuscript. Photoconversion of mEos-PIP5KB from the green (516 nm λ_peak_) to the red fluorescent (580 nm λ_peak_) state was accomplished by exposing cells in the desired field of view to a 50 ms pulse of 0.5 to 1 mW of 405 nm light. The intensity of 405 nm light was measured through the objective using a Newport light meter. Following photoconversion, single cells were imaged for 30 to 60 s with a time resolution of 52 to 102 ms to visualize the membrane-binding dynamics of mEos-PIP5KB. To visualize the localization of mEos-PIP5KB (W365P), we imaged with 52 ms intervals (19 fps), which allowed us to more easily detect transient membrane interactions and rapid diffusion (see Fig. 1, *H*–*K*). To visualize the localization of mEos-PIP5KB (K205, R211A), we imaged with 102 ms intervals (10 fps), which allowed us capture longer time scale differences in membrane dwell times than WT mEos-PIP5KB (see Fig. 3, *E*–*H*).

#### Microscope hardware and imaging acquisition

Single molecule imaging experiments were performed on an inverted Nikon Ti2 microscope using a 100× Nikon objective (1.49 NA) oil immersion TIRF objective. The x-axis and y-axis positions were manually controlled using a Nikon-motorized stage and joystick. All images were acquired using an iXion Life 897 EMCCD camera (Andor Technology Ltd). Fluorescently labeled proteins were excited with either a 405 nm, 488 nm, 561 nm, or 637 nm diode laser (OBIS laser diode, Coherent Inc) controlled by a Vortran laser drive with acousto-optic tunable filters control. The power output measured through the objective for single particle imaging was 1 to 3 mW. For dual color imaging of mNG-PIP5K localization during Cy3-PLCδ–monitored PI(4,5)P_2_ synthesis, samples were excited with 1 mW 488 nm and 1 mW 561 nm light, as measured through the objective. Excitation light was passed through the following dichroic filter cubes before illuminating the sample: (1) ZT488/647rpc and (2) ZT561rdc (ET575LP) (Semrock). Fluorescence emission was detected on an ANDOR EMCCD camera position after a Sutter emission filter wheel housing the following emission filters: ET525/50M, ET600/50M, ET700/75M (Semrock). All *in vitro* experiments were performed at room temperature (23 °C). Single molecule imaging in living cells was performed at 37 °C using an OKO Touch heated stage and heated objective collar. Microscope hardware was controlled using Nikon NIS elements.

#### Single particle tracking

Fluorescent protein detection and tracking was performed using the ImageJ/Fiji TrackMate plugin (66). Data in the form of .nd2 files were loaded in ImageJ. Before being analyzed using TrackMate, data brightness was adjusted for molecules to be easily identifiable. TrackMate was then used to identify and track molecular tracks in these steps: Particles were first identified using the LoG detector option based on brightness and signal-to-noise ratio. Once identified, particles were tracked for their full lifetime using the LAP tracker. This LAP tracker follows molecular displacement as a function of time. Particle trajectories were filtered based on Track Start (removed trajectories that began in first frame), Track End (removed trajectories present in last frame), Duration (removed trajectories ≤ 2 frames and singular extra-long tracks), Track displacement (removed immobilized particles displacement <0.1), and X - Y location (removed particles near the edge of the images). The TrackMate output files were analyzed using PRISM 9 (GraphPad) to calculate characteristic dwell times and diffusion coefficients.

Step size distribution of single particle trajectories were plotted in Prism as frequency *versus* step size (μm). For all analysis presented in this manuscript, the bin size for the step size distribution equals 0.01 μm. For curving fitting, the step-size distributions were plotted as probability density *versus* step size (μm). This was achieved by dividing the frequency distribution (*i.e.* y-axis values) by the bin size (0.01 μm). The probability density *versus* step size plots were fit to the following one- or two-species distributions.

Single species model

$$f\left(r\right)=\frac{r}{2D\tau }e^{-\left(\frac{r^{2}}{4D\tau }\right)}$$

Two species model

$$f\left(r\right)=\alpha \frac{r}{2D_{1}\tau }e^{-\left(\frac{r^{2}}{4D_{1}\tau }\right)}+\left(1-\alpha \right)\frac{r}{2D_{2}\tau }e^{-\left(\frac{r^{2}}{4D_{2}\tau }\right)}$$

Variables are defined as the D1 = diffusion coefficient species 1 (μm^2^/sec), D2 = diffusion coefficient species 2 (μm^2^/sec), alpha (τ_1_ = % of species 1, r = step size (μm), τ_2_ = time interval between steps (sec). Final step size distribution plots were generated in PRISM graphing software and using the following equations: (1 species model): f(x) = x/(2∗D1∗t)∗exp(-(x∧2/(4∗D1∗t))), (2 species model): f(x) = alpha∗(x/(2∗D1∗t)∗exp(-(x∧2/(4∗D1∗t)))) + (1-alpha)∗(x/(2∗D2∗t)∗exp(-(x∧2/(4∗D2∗t)))).

To calculate the single molecule dwell times for Cy5-PIP4K2B, Cy5-PIP4K2B(5K-SL), AF647-PIP5K (and mutants), mNG-PIP5K (and mutants), mNG-PLCδ, and mNG-(5K-SL)-PLCδ, we generated a cumulative distribution frequency plot using the frame interval as the bin size (*e.g.* 50 ms). The log_10_(1-CDF) was plotted against the dwell time and fit to either a single or double exponential decay curve.

Single exponential model

$$f\left(t\right)=e^{\left(-x/\tau \right)}$$

Two exponential model

$$f\left(t\right)={\alpha *e}^{\left(-x/\tau 1\right)}{+\left(1-\alpha \right)*e}^{\left(-x/\tau 2\right)}$$

Fitting procedure initiated with a single exponential. In cases of a low-quality single exponential fit, a maximum of two species model was used. For double exponential fit, alpha (α) represents the fraction of fast dissociating population of molecules characterized by the time constant, τ_1_.

#### Image analysis, curve fitting, and statistics

Image analysis was performed on ImageJ/Fiji. Prism 9 (GraphPad) was used for generating plots, curve fitting, and statistics. Single molecule dwell time and step size presented in this manuscript represent combined data from 2 to 3 movies acquired from multiple fields of view for each technical replicate. Single molecule dwell time errors equal SD for ≥ 3 technical replicates. Dwell time distributions and curve fits were typically generated with n = 500 to 3000 particle trajectories from a single movie. Step size distribution plots and curve fits represent 10,000 to 30,000 measured displacements. When fitting single particle dwell time distributions derived from the visualization of mEos-PIP5KB (WT and mutants) in HEK293T cells, we generated a single dwell time distribution for all the molecules tracked in a single cell. For single molecule data collected in cells, each data point represents the average dwell time, time constant, or displacement for all the molecules detected in a single cell from a single movie (n = 14–27 cells per condition). *p* values (Figs. 1, *I*–*K* and 3, *F* and *G*) were calculated in Prism 9 using an unpaired two-tailed Student’s *t* test. In all cases, the distributions were Gaussian and had similar SDs for comparison.

## Data availability

All the information needed for interpretation of the data is presented in the manuscript or the supplemental material. Plasmids related to this work are available upon request.

## Supporting information

This article contains supporting information (33, 34).

## Conflicts of interest

The authors declare that they have no conflicts of interest with the contents of this article.
