# Supplementary material for: Molecular basis of product recognition during PIP5K-mediated production of PI(4,5)P2 with positive feedback
Source: J Biol Chem. 2024 Aug 3;300(9):107631. doi: 10.1016/j.jbc.2024.107631 (PMC11405805; doi:10.1016/j.jbc.2024.107631)
Supplement: Supplemental Information [file mmc1.docx]

**SUPPLEMENTAL INFORMATION**

**Molecular basis of product recognition during PIP5K-mediated production of PI(4,5)P_2_ with positive feedback**

Benjamin R. Duewell^1,2^, Katherine A. Faris^1,2^, Scott D. Hansen^1,2^*

^1^ Department of Chemistry and Biochemistry, University of Oregon, Eugene, OR 97403

^2^ Institute of Molecular Biology, University of Oregon, Eugene, OR 97403

Corresponding author:

* shansen5@uoregon.edu

**
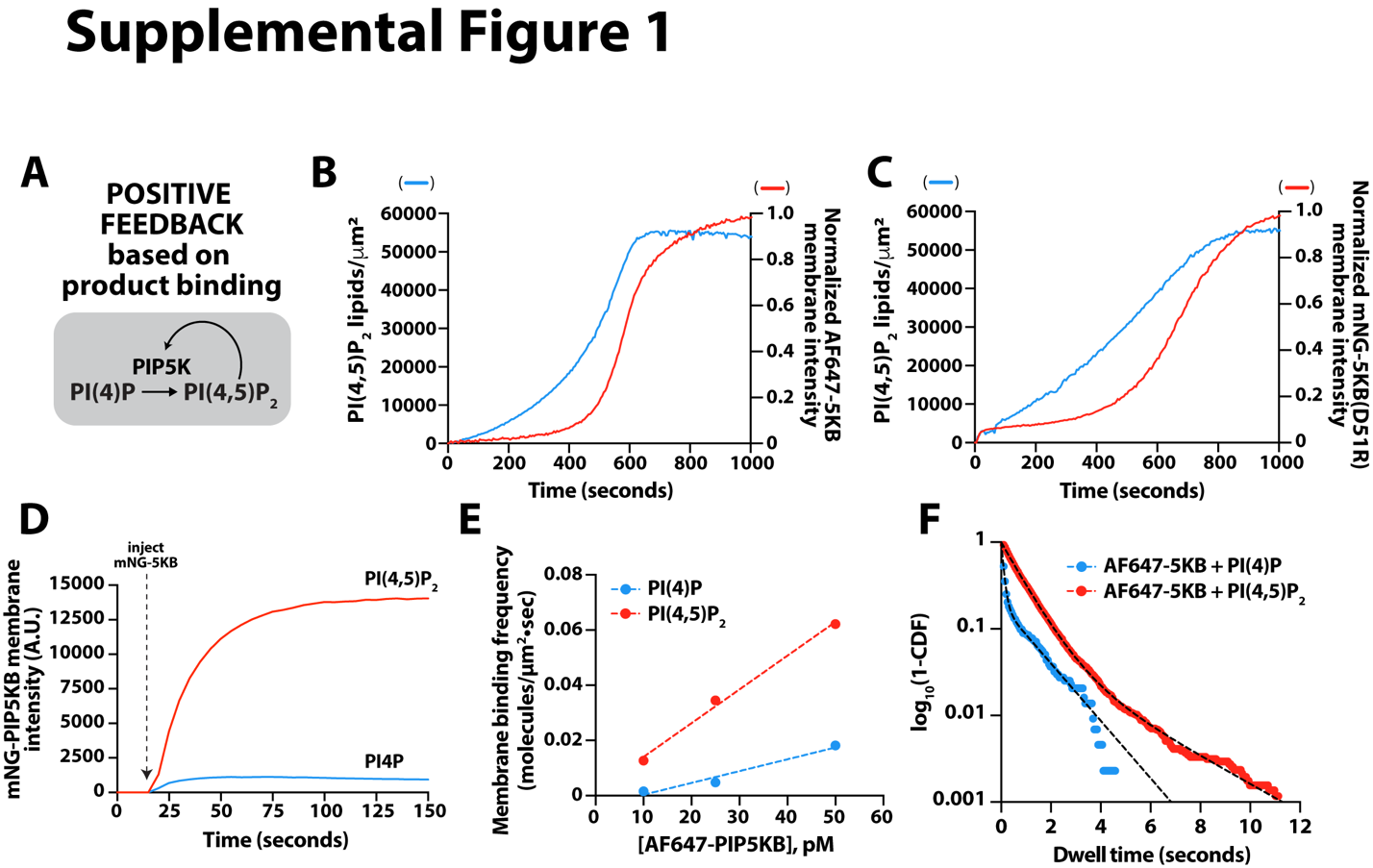
**

**Supplemental Figure 1**

**PIP5KB displays positive feedback based on PI(4,5)P_2_ binding**

**(A)** PIP5K catalyzes the phosphorylation of PI(4)P to generate PI(4,5)P2. Product binding enhances PIP5K membrane localization to drive positive feedback. **(B)** Kinase activity (blue) measures the formation of PI(4,5)P_2_ over time by 5 nM AF647-PIP5KB, while simultaneously tracking normalized AF647-PIP5KB membrane localization (red). **(C)** Kinase activity trace (blue) measures the formation of PI(4,5)P_2_ over time by 5 nM mNG-PIP5KB (D51R), while simultaneously tracking mNG-PIP5KB (D51R) localization (red). **(B-C)** Data used to generate plot in **Figure 1C**. Production of PI(4,5)P_2_ was monitored in the presence of 20 nM Cy3-PLC𝛿. Starting membrane composition: 96% DOPC, 4% PI(4)P. Starting bilayer composition: 96% DOPC, 4% PI(4)P. **(C)** Bulk membrane recruitment of 25 nM mNG-PIP5KB on bilayers containing either 4% PI(4,5)P_2_ or 4% PI(4)P. **(E)** Experimental measurement of membrane binding frequency measured in the presence of 10-50 pM mNG-PIP5KB on bilayers containing either 4% PI(4,5)P_2_ (*k_ON_* = 1.23 nM^-1^•µm^-2^•sec^-1^) or 4% PI(4)P (*k_ON_* = 0.43 nM^-1^•µm^-2^•sec^-1^). **(F)** Representative single molecule dwell time distributions measured in the presence of 10 pM AF647-PIP5KB binding to 96% DOPC bilayers containing either 4% PI(4,5)P_2_ (τ_1_ = 0.82 ± 0.05s, τ_2_ = 3.1 ± 0.34s, α = 0.95 ± 0.01, n = 14950 molecules) or 100 pM AF647-PIP5KB binding to 4% PI4P (τ_1_ = 0.13 ± 0.03s, τ_2_ = 1.64 ± 0.3s, α = 0.88 ± 0.07, n = 3522 molecules). **(D-F)** Imaging buffer contained 1 mM non-hydrolyzable ATPγS to prevent the generation of PI(4,5)P_2_ by PIP5KB. Errors equal SD from n = 3 technical replicates.

**
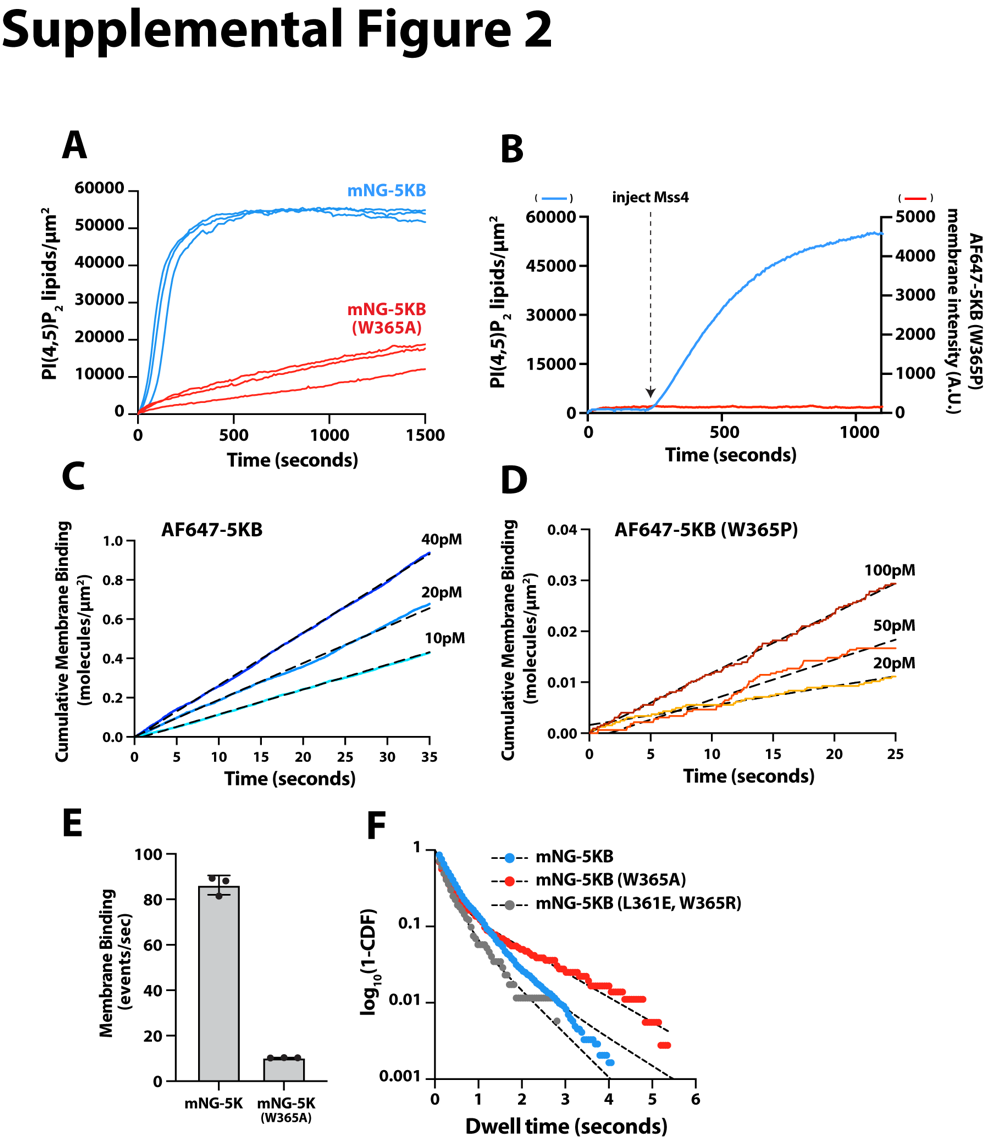
**

**Supplemental Figure 2**

**PIP5KB specificity loop mutants have reduced kinase activity and PI(4,5)P_2_ binding**

**(A)** Lipid kinase activity measured in the presence of 5 nM PIP5KB or 5 nM PIP5KB (W365A). Production of PI(4,5)P_2_ was monitored in the presence of 20 nM Cy3-PLC𝛿. Initial lipid composition: 96% DOPC, 4% PI(4)P. **(B)** Kinase activity trace (blue) measures the formation of PI(4,5)P_2_ over time by 5 nM Mss4, while simultaneously visualizing AF647-PIP5KB (W365P) localization (red). Production of PI(4,5)P_2_ was monitored in the presence of 20 nM Cy3-PLC𝛿. Data used to generate plot in **Figure 1C**. Initial lipid composition: 96% DOPC, 4% PI(4)P. **(C)** Cumulative binding events/time for AF647-PIP5KB WT at concentrations of 10, 20, 40 pM on SLBs containing 96% DOPC and 4% PI(4,5)P_2_ lipids. **(D)** Cumulative binding events/time for AF647-PIP5KB (W365P) at concentrations of 20, 50, and 100 pM on SLBs containing 96% DOPC and 4% PI(4,5)P_2_ lipids. **(C-D)** Data link to **Figure 1E**. **(E)** Membrane binding frequency (events/second) in the presence of 100 pM mNG-PIP5KB (mean = 90.6 ± 3.3 events/sec, *N* = 3 exp) or 100 pM mNG-PIP5KB(W365A) (mean = 10.2 ± 0.03 events/sec, *N* = 3 exp). **(F)** Representative single molecule dwell time distributions measured in the presence of 100 pM mNG-PIP5KB (τ_1_ = 0.43 ± 0.04 s, τ_2_ = 1.07 ± 0.25 s, α = 0.81 ± 0.09, n = 1202), 100 pM mNG-PIP5KB(W365A) (τ_1_ = 0.21 ± 0.01 s, τ_2_ = 1.24 ± 0.01 s, α = 0.89 ± 0.11, n = 1100), or 100 pM mNG-PIP5KB(L361E, W365R) (τ_1_ = 0.21 ± 0.004 s, τ_2_ = 1.41 ± 0.63 s, α = 0.86 ± 0.05, n = 1169). Membrane composition: 98% DOPC, 2% PI(4,5)P_2_ lipids. Errors equal SD from n ≥ 3 technical replicates.

**
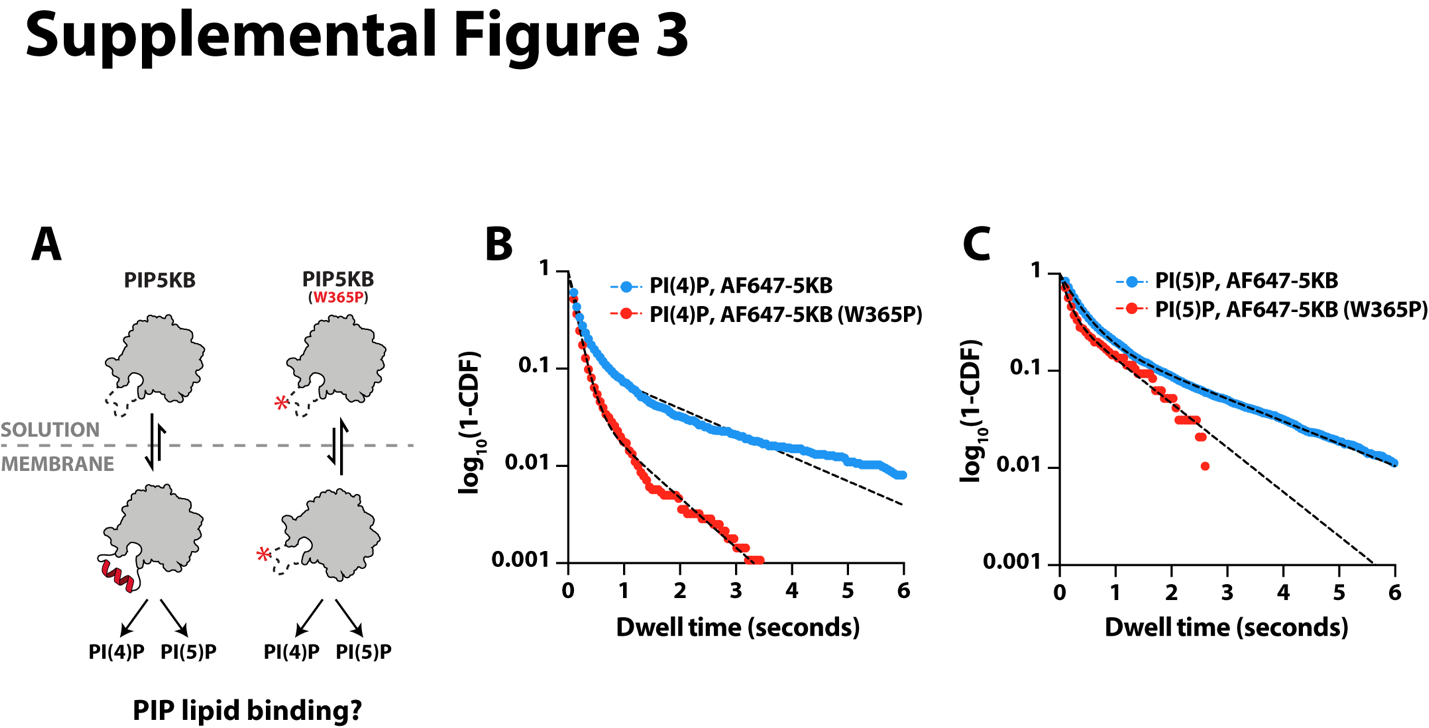
**

**Supplemental Figure 3**

**PIP5KB exhibits specificity for PI(4)P based on single molecule dwell time analysis**

**(A)** Cartoon illustrating the difference of structural organization of PIP5KB, wild type and W365P, in solution versus bound to a lipid bilayer. **(B)** Single molecule dwell time distributions measured in the presence of either 100 pM AF647-PIP5KB (τ_1_ = 0.128 ± 0.03 s, τ_2_ = 1.64 ± 0.3s, α = 0.88 ± 0.07, n = 3522) or 1 nM AF647-PIP5KB(W365P) (τ_1_ = 0.159 ± 0.03 s, τ_2_ = 0.894 ± 0.06 s, α = 0.91 ± 0.06, n = 4055) binding to 96% DOPC SLBs containing 4% PI(4)P. **(C)** Single molecule dwell time distributions measured in the presence of either 100 pM AF647-PIP5KB (τ_1_ = 0.37 ± 0.05 s, τ_2_ = 1.72 ± 0.4 s, α = 0.79 ± 0.07, n = 7526) or 1 nM AF647-PIP5KB(W365P) (τ_1_ = 0.153 s, τ_2_ = 0.954 s, α = 0.62, n = 190) binding to 96% DOPC SLBs containing 4% PI(5)P. Imaging buffer contained 1 mM non-hydrolyzable ATPγS to prevent the generation of PI(4,5)P_2_ by PIP5KB. Errors equal SD n = 2-3 technical replicates.

**
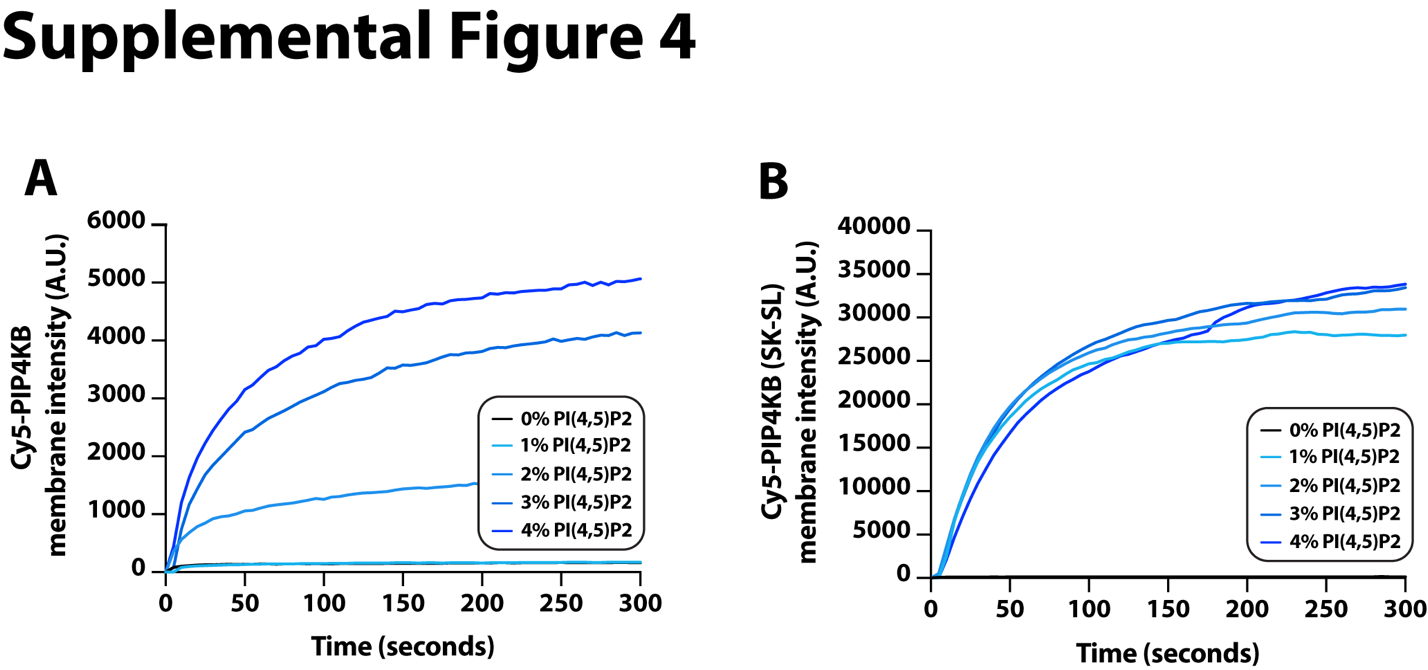
**

**Supplemental Figure 4**

**Membrane binding traces for Cy5-PIP4K2B and Cy5-PIP4K2B (5K-SL) mutant**

**(A)** Raw membrane localization data of 50 nM Cy5-PIP4K2B WT on SLBs containing 0, 1, 2, 3, and 4% PI(4,5)P_2_ lipids. **(B)** Raw membrane localization data of 50 nM Cy5-PIP4K2B (5K-SL) on SLBs containing 0, 1, 2, 3, and 4% PI(4,5)P_2_ lipids. **(A-B)** Graphs correspond to the data plotted in Figure 2F.

**PLASMID INVENTORY**

| **Recombinant DNA** | | | |
| --- | --- | --- | --- |
| his6-TEV-SUMO3-GGGGG-PLCδ PH domain (11-140aa) | bacterial | Hansen et al. 2019 | pSH450 |
| his6-MBP-TEV-GGGGG-PIP5KB (1-421aa) | baculovirus | Hansen et al. 2019 | pSH495 |
| his6-MBP-TEV-GGGGG-PIP5KB (1-421aa) W365P | baculovirus | This paper | pSH808 |
| his6-MBP-TEV-GGGGG-PIP5KB (1-421aa) K205A, R211A | baculovirus | This paper | pSH902 |
| his6-TEV-mNG-PIP5KB (1-421aa) | baculovirus | Hansen et al. 2022 | pSH908 |
| his6-TEV-mNG-PIP5KB (1-421aa) D51R | baculovirus | Hansen et al. 2022 | pSH1271 |
| his6-TEV-mNG-PIP5KB (1-421aa) K138A | baculovirus | This paper | pSH1349 |
| his6-TEV-mNG-PIP5KB (1-421aa) D266K | baculovirus | This paper | pSH1350 |
| his6-TEV-mNG-PIP5KB (1-421aa) D350A | baculovirus | This paper | pSH1352 |
| his6-TEV-SpyTag-mNG-PIP5KB (1-421aa) K205A, R211A | baculovirus | This paper | pSH1318 |
| his10-TEV-mNG-5KB SL (355-377aa)-PLCδ PH (11-140aa) | bacterial | This paper | pSH1239 |
| his10-TEV-mNG-PLCδ PH domain (11-140aa) | bacterial | This paper | pSH1270 |
| his6-TEV-SUMO3-GGGG-PIP4K2B (native specificity loop) | bacterial | This paper | pSH455 |
| his6-TEV-SUMO3-GGGG-PIP4K2B (5KB specificity loop) | bacterial | This paper | pSH481 |
| pHS-mEos3.2-PIP5KB (1-421aa) | mammalian | This paper | pSH787 |
| pHS-mEos3.2-PIP5KB (1-421aa) W365P | mammalian | This paper | pSH1281 |
| pHS-mEos3.2-PIP5KB (1-421aa) K205A, R211A | mammalian | This paper | pSH1338 |
| pSFFV-mNG-(GGGGS)x2-PIP5KB (1-421aa) | mammalian | This paper | pSH887 |
| pSFFV-mNG-(GGGGS)x2-PIP5KB (1-421aa) W365A | mammalian | This paper | pSH1417 |
| pSFFV-mNG-(GGGGS)x2-PIP5KB (1-421aa) L61E, W365R | mammalian | This paper | pSH1418 |

**PEPTIDE SEQUENCES**

The protein sequences of recombinantly expressed and purified proteins used in the study are shown below. The following tags were cleaved off the recombinant proteins and removed during the purification:

**his6-TEV-SUMO3 (**↓= site of SUMO protease cleavage)

MKHHHHHHPMSDYDIPTTENLYFQGAMGNDHINLKVAGQDGSVVQFKIKRHTPLSKLMKAYCERQGLSMRQIRFRFDGQPINETDTPAQLEMEDEDTIDVFQQQTGG↓GGGG

**his6-MBP-N10-TEV (**↓= site of TEV protease cleavage)

MGSSHHHHHHGSSMKIEEGKLVIWINGDKGYNGLAEVGKKFEKDTGIKVTVEHPDKLEEKFPQVAATGDGPDIIFWAHDRFGGYAQSGLLAEITPDKAFQDKLYPFTWDAVRYNGKLIAYPIAVEALSLIYNKDLLPNPPKTWEEIPALDKELKAKGKSALMFNLQEPYFTWPLIAADGGYAFKYENGKYDIKDVGVDNAGAKAGLTFLVDLIKNKHMNADTDYSIAEAAFNKGETAMTINGPWAWSNIDTSKVNYGVTVLPTFKGQPSKPFVGVLSAGINAASPNKELAKEFLENYLLTDEGLEAVNKDKPLGAVALKSYEEELAKDPRIAATMENAQKGEIMPNIPQMSAFWYAVRTAVINAASGRQTVDEALKDAQTNSSSNNNNNNNNNNLGIEENLYFQ↓GGGGG

**his6-TEV (**↓= site of TEV protease cleavage)

MGSSHHHHHHENLYFQ↓SN

**his10-TEV (**↓= site of TEV protease cleavage)

MKHHHHHHHHHHYDIPTTENLYFQ↓GA

Shown below are the plasmid #’s and names of the full-length recombinant proteins that were expressed and purified in this study. The affinity and solubility tags that are underlined in the gene names were cleaved off the indicated protein and removed through purification. The primary amino acid sequence shown below represents the final purified product.

**pSH450, his6-TEV-SUMO3-GGGG-PLCδ PH domain (11-140aa)**

…GGGGTSHGLQDDEDLQALLKGSQLLKVKSSSWRRERFYKLQEDCKTIWQESRKVMRTPESQLFSIEDIQEVRMGHRTEGLEKFARDVPEDRCFSIVFKDQRNTLDLIAPSPADAQHWVLGLHKIIHHSGSMDQRQKGS*

**pSH455, his6-TEV-SUMO3-GGGG-PIP4K2B (native specificity loop)**

**…**GGGGTSMSSNCTSTTAVAVAPLSASKTKTKKKHFVCQKVKLFRASEPILSVLMWGVNHTINELSNVPVPVMLMPDDFKAYSKIKVDNHLFNKENLPSRFKFKEYCPMVFRNLRERFGIDDQDYQNSVTRSAPINSDSQGRCGTRFLTTYDRRFVIKTVSSEDVAEMHNILKKYHQFIVECHGNTLLPQFLGMYRLTVDGVETYMVVTRNVFSHRLTVHRKYDLKGSTVAREASDKEKAKDLPTFKDNDFLNEGQKLHVGEESKKNFLEKLKRDVEFLAQLKIMDYSLLVGIHDVDRAEQEEMEVEERAEDEECENDGVGGNLLCSYGTPPDSPGNLLSFPRFFGPGEFDPSVDVYAMKSHESSPKKEVYFMAIIDILTPYDTKKKAAHAAKTVKHGAGAEISTVNPEQYSKRFNEFMSNILT*

**pSH481, his6-TEV-SUMO3-GGGG-PIP4K2B (containing the PIP5KB specificity loop)**

**…**GGGGTSMSSNCTSTTAVAVAPLSASKTKTKKKHFVCQKVKLFRASEPILSVLMWGVNHTINELSNVPVPVMLMPDDFKAYSKIKVDNHLFNKENLPSRFKFKEYCPMVFRNLRERFGIDDQDYQNSVTRSAPINSDSQGRCGTRFLTTYDRRFVIKTVSSEDVAEMHNILKKYHQFIVECHGNTLLPQFLGMYRLTVDGVETYMVVTRNVFSHRLTVHRKYDLKGSTVAREASDKEKAKDLPTFKDNDFLNEGQKLHVGEESKKNFLEKLKRDVEFLAQLKIMDYSLLVGIHDVDRAEQEEMEVEERAEDEECENDGVGGNLLCSYGTPPDSPGNLLSFPRFFGPGEFDPSVDVYAMKSHESSPKKEVYFMAIIDILTPYRLMKKLEHSWKALVYDGDTVSTVNPEQYSKRFNEFMSNILT*

**pSH495, his6-MBP-N10-TEV-GGGG-PIP5KB (1-421aa)**

…GGGGGMSSAAENGEAAPGKQNEEKTYKKTASSAIKGAIQLGIGYTVGNLTSKPERDVLMQDFYVVESVFLPSEGSNLTPAHHYPDFRFKTYAPLAFRYFRELFGIKPDDYLYSICSEPLIELSNPGASGSLFFVTSDDEFIIKTVQHKEAEFLQKLLPGYYMNLNQNPRTLLPKFYGLYCMQSGGINIRIVVMNNVLPRSMRMHFTYDLKGSTYKRRASRKEREKSNPTFKDLDFLQDMHEGLYFDTETYNALMKTLQRDCRVLESFKIMDYSLLLGIHFLDHSLKEKEEETPQNVPDAKRTGMQKVLYSTAMESIQGPGKSGDGIITENPDTMGGIPAKSHRGEKLLLFMGIIDILQSYRLMKKLEHSWKALVYDGDTVSVHRSFYADRFLKFMNSRVFKKIQALKASPSKKRCNSIAALKATS*

**pSH787, pHS-mEos3.2-PIP5KB (1-421aa)**

MSAIKPDMKIKLRMEGNVNGHHFVIDGDGTGKPFEGKQSMDLKVKEGGPLPFAFDILTTAFHYGNRVFAKYPDNIQDYFKQSFPKGYSWERSLTFEDGGICNARNDITMEGDTFYNKVRFYGTNFPANGPVMQKKTLKWEPSTEKMYVRDGVLTGDIEMALLLEGNAHYRCDFRTTYKAKEKGVKLPGAHFVDHCIEILSHDKDYNKVKLYEHAVAHSGLPDNARRGGGGSGGGGSMSSAAENGEAAPGKQNEEKTYKKTASSAIKGAIQLGIGYTVGNLTSKPERDVLMQDFYVVESVFLPSEGSNLTPAHHYPDFRFKTYAPLAFRYFRELFGIKPDDYLYSICSEPLIELSNPGASGSLFFVTSDDEFIIKTVQHKEAEFLQKLLPGYYMNLNQNPRTLLPKFYGLYCMQSGGINIRIVVMNNVLPRSMRMHFTYDLKGSTYKRRASRKEREKSNPTFKDLDFLQDMHEGLYFDTETYNALMKTLQRDCRVLESFKIMDYSLLLGIHFLDHSLKEKEEETPQNVPDAKRTGMQKVLYSTAMESIQGPGKSGDGIITENPDTMGGIPAKSHRGEKLLLFMGIIDILQSYRLMKKLEHSWKALVYDGDTVSVHRPSFYADRFLKFMNSRVFKKIQALKASPSKKRCNSIAALKATS*

**pSH808, his6-MBP-N10-TEV-GGGGG-PIP5KB (1-421aa) W395P**

…GGGGGMSSAAENGEAAPGKQNEEKTYKKTASSAIKGAIQLGIGYTVGNLTSKPERDVLMQDFYVVESVFLPSEGSNLTPAHHYPDFRFKTYAPLAFRYFRELFGIKPDDYLYSICSEPLIELSNPGASGSLFFVTSDDEFIIKTVQHKEAEFLQKLLPGYYMNLNQNPRTLLPKFYGLYCMQSGGINIRIVVMNNVLPRSMRMHFTYDLKGSTYKRRASRKEREKSNPTFKDLDFLQDMHEGLYFDTETYNALMKTLQRDCRVLESFKIMDYSLLLGIHFLDHSLKEKEEETPQNVPDAKRTGMQKVLYSTAMESIQGPGKSGDGIITENPDTMGGIPAKSHRGEKLLLFMGIIDILQSYRLMKKLEHSPKALVYDGDTVSVHRSFYADRFLKFMNSRVFKKIQALKASPSKKRCNSIAALKATS*

**pSH902, his6-MBP-N10-TEV-GGGGG-PIP5KB (1-421aa) K205A, R211A**

…GGGGGTSMSSAAENGEAAPGKQNEEKTYKKTASSAIKGAIQLGIGYTVGNLTSKPERDVLMQDFYVVESVFLPSEGSNLTPAHHYPDFRFKTYAPLAFRYFRELFGIKPDDYLYSICSEPLIELSNPGASGSLFFVTSDDEFIIKTVQHKEAEFLQKLLPGYYMNLNQNPRTLLPKFYGLYCMQSGGINIRIVVMNNVLPRSMRMHFTYDLAGSTYKARASRKEREKSNPTFKDLDFLQDMHEGLYFDTETYNALMKTLQRDCRVLESFKIMDYSLLLGIHFLDHSLKEKEEETPQNVPDAKRTGMQKVLYSTAMESIQGPGKSGDGIITENPDTMGGIPAKSHRGEKLLLFMGIIDILQSYRLMKKLEHSWKALVYDGDTVSVHRPSFYADRFLKFMNSRVFKKIQALKASPSKKRCNSIAALKATS*

**pSH908, his6-TEV-mNeonGreen-(GGGGS)x2-PIP5KB (1-421aa)**

**…**SNTGMVSKGEEDNMASLPATHELHIFGSINGVDFDMVGQGTGNPNDGYEELNLKSTKGDLQFSPWILVPHIGYGFHQYLPYPDGMSPFQAAMVDGSGYQVHRTMQFEDGASLTVNYRYTYEGSHIKGEAQVKGTGFPADGPVMTNSLTAADWCRSKKTYPNDKTIISTFKWSYTTGNGKRYRSTARTTYTFAKPMAANYLKNQPMYVFRKTELKHSKTELNFKEWQKAFTDVMGMDELYKGGGGSGGGGSTSMSSAAENGEAAPGKQNEEKTYKKTASSAIKGAIQLGIGYTVGNLTSKPERDVLMQDFYVVESVFLPSEGSNLTPAHHYPDFRFKTYAPLAFRYFRELFGIKPDDYLYSICSEPLIELSNPGASGSLFFVTSDDEFIIKTVQHKEAEFLQKLLPGYYMNLNQNPRTLLPKFYGLYCMQSGGINIRIVVMNNVLPRSMRMHFTYDLKGSTYKRRASRKEREKSNPTFKDLDFLQDMHEGLYFDTETYNALMKTLQRDCRVLESFKIMDYSLLLGIHFLDHSLKEKEEETPQNVPDAKRTGMQKVLYSTAMESIQGPGKSGDGIITENPDTMGGIPAKSHRGEKLLLFMGIIDILQSYRLMKKLEHSWKALVYDGDTVSVHRPSFYADRFLKFMNSRVFKKIQALKASPSKKRCNSIAALKATS*

**pSH1239, his10-TEV-mNG-5KB Specificity loop (355-377aa)-PLCδ PH (11-140aa)**

MKHHHHHHHHHHYDIPTTENLYFQGAMVSKGEEDNMASLPATHELHIFGSINGVDFDMVGQGTGNPNDGYEELNLKSTKGDLQFSPWILVPHIGYGFHQYLPYPDGMSPFQAAMVDGSGYQVHRTMQFEDGASLTVNYRYTYEGSHIKGEAQVKGTGFPADGPVMTNSLTAADWCRSKKTYPNDKTIISTFKWSYTTGNGKRYRSTARTTYTFAKPMAANYLKNQPMYVFRKTELKHSKTELNFKEWQKAFTDVMGMDELYKSLVQSYRLMKKLEHSWKALVYDGDTVHGLQDDEDLQALLKGSQLLKVKSSSWRRERFYKLQEDCKTIWQESRKVMRTPESQLFSIEDIQEVRMGHRTEGLEKFARDVPEDRCFSIVFKDQRNTLDLIAPSPADAQHWVLGLHKIIHHSGSMDQRQKGS*

**pSH1270, his10-TEV-mNG-PLCδ PH domain (11-140aa)**

MKHHHHHHHHHHYDIPTTENLYFQGAMVSKGEEDNMASLPATHELHIFGSINGVDFDMVGQGTGNPNDGYEELNLKSTKGDLQFSPWILVPHIGYGFHQYLPYPDGMSPFQAAMVDGSGYQVHRTMQFEDGASLTVNYRYTYEGSHIKGEAQVKGTGFPADGPVMTNSLTAADWCRSKKTYPNDKTIISTFKWSYTTGNGKRYRSTARTTYTFAKPMAANYLKNQPMYVFRKTELKHSKTELNFKEWQKAFTDVMGMDELYKHGLQDDEDLQALLKGSQLLKVKSSSWRRERFYKLQEDCKTIWQESRKVMRTPESQLFSIEDIQEVRMGHRTEGLEKFARDVPEDRCFSIVFKDQRNTLDLIAPSPADAQHWVLGLHKIIHHSGSMDQRQKGS*

**pSH1318, his6-TEV-SpyTag-mNG-PIP5KB (1-421aa) K205A, R211A**

**…**NAHIVMVDAYKPTKTGMVSKGEEDNMASLPATHELHIFGSINGVDFDMVGQGTGNPNDGYEELNLKSTKGDLQFSPWILVPHIGYGFHQYLPYPDGMSPFQAAMVDGSGYQVHRTMQFEDGASLTVNYRYTYEGSHIKGEAQVKGTGFPADGPVMTNSLTAAWCRSKKTYPNDKTIISTFKWSYTTGNGKRYRSTARTTYTFAKPMAANYLKNQPMYVFRKTELKHSKTELNFKEWQKAFTDVMGMDELYKGGGGSGGGGSTSMSSAAENGEAAPGKQNEEKTYKKTASSAIKGAIQLGIGYTVGNLTSKPERDVLMQDFYVVESVFLPSEGSNLTPAHHYPDFRFKTYAPLAFRYFRELFGIKPDDYLYSICSEPLIELSNPGASGSLFFVTSDDEFIIKTVQHKEAEFLQKLLPGYYMNLNQNPRTLLPKFYGLYCMQSGGINIRIVVMNNVLPRSMRMHFTYDLAGSTYKARASRKEREKSNPTFKDLDFLQDMHEGLYFDTETYNALMKTLQRDCRVLESFKIMDYSLLLGIHFLDHSLKEKEEETPQNVPDAKRTGMQKVLYSTAMESIQGPGKSGDGIITENPDTMGGIPAKSHRGEKLLLFMGIIDILQSYRLMKKLEHSWKALVYDGDTVSVHRPSFYADRFLKFMNSRVFKKIQALKASPSKKRCNSIAALKATS*
